# Mitochondrial genome and its regulator TFAM modulates head and neck tumourigenesis through intracellular metabolic reprogramming and activation of oncogenic effectors

**DOI:** 10.1038/s41419-021-04255-w

**Published:** 2021-10-18

**Authors:** Yi-Ta Hsieh, Hsi-Feng Tu, Muh-Hwa Yang, Yi-Fen Chen, Xiang-Yun Lan, Chien-Ling Huang, Hsin-Ming Chen, Wan-Chun Li

**Affiliations:** 1 Institute of Oral Biology, College of Dentistry, National Yang Ming Chiao Tung University, Taipei, Taiwan; 2 Department of Dentistry, College of Dentistry, National Yang Ming Chiao Tung University, Taipei, Taiwan; 3 Department of Dentistry, National Yang Ming Chiao Tung University Hospital, Yilan, Taiwan; 4 Institute of Clinical Medicine, National Yang Ming Chiao Tung University, Taipei, Taiwan; 5 Division of Medical Oncology, Taipei Veterans General Hospital, Taipei, Taiwan; 6 Cancer Progression Research Center, National Yang Ming Chiao Tung University, Taipei, Taiwan; 7 Department of Health Technology and Informatics (HTI), The Hong Kong Polytechnic University (PolyU), Hung Hom, Kowloon, Hong Kong, SAR China; 8 School of Dentistry and Department of Dentistry, National Taiwan University Medical College and National Taiwan University Hospital, Taipei, Taiwan

**Keywords:** Cancer metabolism, Oral cancer

## Abstract

Mitochondrial transcriptional factor A (TFAM) acts as a key regulatory to control mitochondrial DNA (mtDNA); the impact of TFAM and mtDNA in modulating carcinogenesis is controversial. Current study aims to define TFAM mediated regulations in head and neck cancer (HNC). Multifaceted analyses in HNC cells genetically manipulated for TFAM were performed. Clinical associations of TFAM and mtDNA encoded Electron Transport Chain (ETC) genes in regulating HNC tumourigenesis were also examined in HNC specimens. At cellular level, TFAM silencing led to an enhanced cell growth, motility and chemoresistance whereas enforced TFAM expression significantly reversed these phenotypic changes. These TFAM mediated cellular changes resulted from (1) metabolic reprogramming by directing metabolism towards aerobic glycolysis, based on the detection of less respiratory capacity in accompany with greater lactate production; and/or (2) enhanced ERK1/2-Akt-mTORC-S6 signalling activity in response to TFAM induced mtDNA perturbance. Clinical impacts of TFAM and mtDNA were further defined in carcinogen-induced mouse tongue cancer and clinical human HNC tissues; as the results showed that TFAM and mtDNA expression were significantly dropped in tumour compared with their normal counterparts and negatively correlated with disease progression. Collectively, our data uncovered a tumour-suppressing role of TFAM and mtDNA in determining HNC oncogenicity and potentially paved the way for development of TFAM/mtDNA based scheme for HNC diagnosis.

## Introduction

The progression of malignant transformation from non-neoplastic cells to tumourous cells can be achieved only by contribution of a series of cellular events [1]. In the aspect of energy production, it is widely accepted that, when compared to normal cells, cancer cells are more prone to consume greater glucose thereby forming lactate by a glycolytic pathway regardless of oxygen availability [2]. Indeed, a number of studies have shown that enzymes involved in central carbon metabolism including glycolysis and Pentose Phosphate Pathway (PPP) as well as *de novo* lipogenesis and glutaminolysis are enhanced in tumours compared with their normal counterparts; the mitochondrial related metabolic activity, on the other hand, is often downregulated [3–6].

To date, most investigations focus on the role of glycolytic enzymes in regulating tumourigenesis [7, 8], mainly due to the fact that a molecular basis differential expression of glycolytic enzymes and glucose transporters has been shown to correlate with mutations of a classic tumour suppressor protein TP53 in most cancers [9]. The roles of mitochondrial cues in controlling cellular malignancy, however, have been under appreciated. For example, mitochondrial aldehyde dehydrogenase (ALDH) could facilitate cytosolic NADH content which serves as an electron donor to trigger ATP production, thereby promoting tumour progression [10]. A high level of glutamine transporter (SLC1A5) and glutaminases (GLS), two key factors involved in the conversion of glutamine into glutamate supplying nitrogen for anaplerotic flux to TriCarboxylic Acid (TCA) cycle, was detected in c-myc driven cancers [11]. Interestingly, enhanced Electron Transport Chain (ETC) activity was found in Venetoclax-resistant multiple myeloma cells while application of ETC inhibitor IACS-010759 and thenoyltrifluoroacetone (TTFA) could sensitize resistance to Venetoclax through the ATF4-BIM-NOXA pathway [12] indicating that mitochondrial activity may not necessarily be downregulated during tumourigenesis. In addition to metabolic enzymes or transporters, accumulating “oncometabolites” due to defective TCA cycle enzymes, such as fumarate hydratase (FH), succinate dehydrogenase (SDH) and isocitrate dehydrogenase (IDH), were also detected in tumour cells implying that deregulated mitochondrial metabolites might also underlie oncogenic transformation [13, 14].

Mitochondria are a double-membrane organelle that contributes to energy production in most eukaryotic organisms via various bioenergetic processes including the TCA cycle, ETC and lipid catabolism. A variety of cellular processes such as cell apoptosis, differentiation, calcium homeostasis and hormone biosynthesis occur in different mitochondrial compartments [15, 16]. With distinct structural and physiological functions, mitochondria are interestingly regarded as an independent “organism” in a cell based on the fact that mitochondria have their own genome, named mitochondrial DNA (mtDNA) [17], and that mitochondria could be horizontally transferred between cells resulting in different pathophysiological consequences [18]. In humans, mtDNA is a circular genomic material and encodes 13 ETC protein subunits [17]. At the molecular level, mitochondrial transcription factor A (TFAM) plays a key role in controlling mtDNA packing, replication, and transcription [17]. In clinical studies, lower mtDNA and TFAM is associated with poorer survival in ovarian cancer [19], oesophageal squamous cell carcinoma [20], colorectal cancer [21] as well as oral squamous cell carcinoma [22]. At cellular basis, decreased TFAM and mtDNA content led to lower mitochondrial activity resulting in greater lactate production, increased cell proliferation and enhanced metastatic capacity in breast cancer [23], intestinal cancer [24], oesophageal cancer [20], and cisplatin (CDDP) resistant ovarian cancer [19]. A more recent study showed that TFAM mediated regulations for oncogenicity in various cancers probably occur via the disruption of LC3-II-mediated autophagy [25]. In brief, most studies suggest that impaired mitochondrial could accelerate oncogenicity in different cancers, both in vitro and in vivo.

Until now, an impact of TFAM/mtDNA in modulating cellular, molecular and metabolic identity of Head and Neck Cancer (HNC) remains elusive. Our recent study demonstrated that manipulation of a rate-limiting factor of Pyruvate Dehydrogenase complex (PDC), and Pyruvate Dehydrogenase E1 subunit (PDHA1) that controls the metabolic fate of lactate entering into mitochondria, could sufficiently enhance HNC cellular malignancy [26]. This is interesting to further verify the role of mitochondrial genome and mtDNA regulator TFAM in controlling HNC malignancy.

## Material and methods

### Chemical, cell culture, animal and clinical tissues

Puromycin, 3-(4,5-Dimethylthiazol-2-yl)-2,5-diphenyltetrazolium bromide (MTT), CDDP, 5-fluouracil (5-FU), and Extracellular signal-regulated kinase 1/2 (ERK1/2) inhibitor PD98059 were purchased from Sigma-Aldrich. Protein kinase B (PKB/Akt) inhibitor MK-2206 was obtained from Selleckchem. The source and culture conditions of HNC cells (all are Human PapillomaVirus negative (HPV^−^)) from different origins as well as in HNC bearing xenografic tumour growth assay [under the approval of Institutional Animal Care and Use Committee (IACUC), National Yang Ming Chiao Tung University (NYCU) were described elsewhere [26, 27]. In brief, the same kind of 10^6^ control (shLuc) and TFAM silencing HNC cells were transplanted in opposite side of flank in the same male nude mouse (5–7 weeks old CAnN.Cg-Foxn1nu/CrlNarl from Academia Sinica *N* = 9–10). The tumour size was followed every week and at the 4th week post transplantation, tumours were excised and weighted. Clinical human HNC tumours and its adjacent normal tissues were obtained from the Department of Oral Maxillofacial Surgery, NYCU Hospital under approval of the Institutional Review Boards (IRB) of NYCU Hospital (IRB#: NYMUH 2018B003) and were preserved in RNAlater immediately after surgical resection for qRT-PCR analysis (Table S1).

### Establishment of TFAM deficient and overexpressing HNC cells

Plasmids encoding small hairpin RNAs (shRNA) targeting 3ʹ-untranslated regions (3ʹ-UTR) of TFAM gene (shTFAM) were obtained from the National RNAi Core Facility, Academia Sinica, Taiwan (Table S2). The lentiviral vectors encoding shTFAM and the control shRNA targeting Luciferase (shLuc) were produced in 293 T cells for infection into HNC cells by using gene juice transfection reagent. HSC3 cells encoding clone A shTFAM, OECM1 cells encoding clone B shTFAM and SAS/FaDu cells encoding clone C shTFAM were selected for further experiments based on their knockdown efficiency. Stable knockdown cell lines were maintained in indicated complete medium containing 2.5 μg/ml puromycin. For making the TFAM overexpressing vector, primers targeting wild-type TFAM gene carrying BamHI (5ʹ-CGAGGATCCACCATGGCGTTTCTCCGAAGC-3ʹ) and Notl (5ʹ- GTAGCGGCCGCATACACTCCTCAGCACCATA-3ʹ) restriction enzyme sequences were used to amplify full length TFAM cDNA from SAS cells, which encode wild-type TFAM, using the Platinum PCR SuperMix High Fidelity system (Invitrogen™). The PCR products were then cloned into the pcDNA4/myc-HisA,B,C vector (Thermo Fisher Scientific) between BamHl and NotI. Candidate colonies were selected, amplified (Plasmid Midi kit, Geneaid) and sequence was verified before transfection. For TFAM overexpression in HNC cells, TransFectin™ Lipid Reagent (BIO-RAD) was utilized following the manufacturer’s instructions.

### Determination of half maximal inhibitory concentration (IC50) of chemotherapeutic agents

The assay to define IC50 of different chemotherapeutic agents in HNC cells was performed by analysis for viability of cells treated with serial diluted chemotherapeutic agents. While the inhibitory rates, defined as the percentage of mean OD value in treatment group to the mean OD value in control group plotted versus chemotherapeutic agents concentrations, IC50 could be calculated in Prism 5 using non-linear regression analysis followed by the panel of equations “Dose-response curves––Inhibition” and an equation [log (inhibitor concentration) vs. normalized response].

### Establishment of chemotherapy and PhotoDynamic Therapy (PDT) resistance HNC cells

The CDDP resistant, 5FU resistant and PDT resistant HNC cells were generated by applying increasing dose of candidate drugs/treatment for multiple selection cycles onto parental HNC cells (sensitive counterpart). In brief, HNC cells were treated with low-dose CDDP and 5FU for 48 h, allowed the cells to recovery up to 70–80% confluency, passaged for two times in the fresh medium without drugs and applied higher dose of drugs for further selection. CDDP and 5FU resistant HNC cells could be obtained after repeating procedure for five times. For establishment of PDT resistant HNC cells, parental cells were pre-treated with 1 mM 5-aminolevulinic acid (ALA) for 3 h followed the irradiation of red light (wave length: 635 ± 5 nm) at different dose in Ca9-22 and SAS cells and 48-h recovery before the next round of treatment. PDT resistant HNC cells could be obtained after repeating procedure for 5 times. The power density was 87 mW/cm^2^ and the period of light treatment was set at 11.49 s to deliver an energy dose of 1 joule(J)/cm^2^ [28]. The established resistant HNC cells were validated for greater IC50 in contrast to their sensitive counterparts.

### In vitro cell-based analysis

Cell growth was measured by both MTT assay and Trypan blue exclusion assay while the cell cycle, Annexin V-FITC based cell apoptosis, transwell-based cell migration, drug resistance assay, quantitative real-time PCR (qRT-PCR), and Western blot analysis was previously described [26]. Primers for qRT-PCR analysis (Table S3) and antibodies used for Western blot and Immunocytochemistry (ICC) analysis are listed (Table S4). For ICC analysis, cells were seeded on 20 × 20 mm cover slides and cultured in indicated medium. The cells cultured on cover slides were fixed with 4% paraformaldehyde (PFA) for 20–30 min and incubated with PBS in 4°C until use. The primary and secondary antibodies were sequentially applied onto the cells and DAPI was used as counter stain to define cell nuclei. Stainings were examined using Olympus FV1000 Confocal microscope or Zeiss LSM880 with AiryScan at Instrumentation Resource Center (IRC), NYCU. Final images were processed using Adobe Photoshop or PhotoImpact X3. Image J was used to quantify protein expression.

### Fluorogenic Caspase-3 activity assay

Cells were seeded in 96-well plate for 24 h, rinse cell with ice-cold PBS and spin the plate at 300xg for 10 min. Cell lysates were collected by cell lysis buffer on ice for further use. Caspase-3 activity was detected following the manufacturer’s instructions (Cell signalling, Kit#5723). N-Acetyl-Asp-Glu-Val-Asp-7-amino-4-methylcoumarin (Ac-DEVD-AMC) is used as the fluorogenic substrate while activated Caspase-3 cleaves this substrate between DEVD and AMC, generating highly fluorescent AMC that is detected using TECAN SPARK multimode microplate readers at IRC, NYCU. Fluorescence intensity was measured with excitation wavelength at 380 nm and emission wavelength at 450 nm at 37 °C.

### Metabolic assays

Lactate colorimetric/fluorometric assay kit, ATP colorimetric/fluorometric Assay kit, Pyruvate assay kit, PDH Activity Colorimetric Assay kit (Biovision) and Glucose Uptake Cell-based Assay kit (Cayman Chemical) were performed following the manufacturer’s instructions. Colorimetric measurements for optical density (OD) and flow cytometrical analysis were performed using TECAN SPARK multimode microplate readers and Beckman Coulter CytoFLEX, respectively, at IRC, NYCU.

### Mitochondrial assays

To determine Oxygen Consumption Rates (OCRs), Seahorse XF bioanalyser was used following the manufacturer’s instructions. The relative mtDNA copy number was measured by qRT-PCR. MitoTracker™ Red CMXRos, Carboxy-DCFDA, MitoSOX™ Red and Tetramethylrhodamine, ethyl ester (Thermo Fisher Scientific) were used to detect mitochondrial mass and activity, respectively, using flow cytometrical analysis by Beckman Coulter CytoFLEX. Living cells were also stained with MitoTracker™ Red CMXRos, followed by fixation of 4% PFA and counterstained for nuclei by DAPI. Images were captured using Zeiss LSM880 with AiryScan at IRC, NYCU. Final images were processed using Adobe Photoshop or PhotoImpact X3.

### Metabolomics analysis

Cellular metabolites were analysed using Liquid Chromatography-Mass Spectrophotometry (LC-MS) at Metabolomics Core laboratory at NYCU. In brief, 10^6^ cells were washed with PBS and treated with LC-MS grade iced methanol and water followed by vigorous vortexing and centrifugation to remove cell debris. The supernatants were collected for LC-MS metabolic profiling analysis.

### RNA sequencing analysis for chemical-induced mouse tongue cancer

For time-course tongue cancer induction, previously used carcinogen 4-nitroquinoline-1-oxide (Nqo, Sigma-Aldrich) was freshly prepared in 1,2-propylene glycol (Sigma-Aldrich) and fed in water (final concentration: 100 μg/ml) for 6–8 weeks old C57BL/6 mice based on protocol described previously [29]. At the indicated time point (8-, 12- and 16-weeks post-treatment), the mice were sacrificed and tongue tissues were harvested and preserved in RNALater for RNA sequencing (RNA-seq) analysis. For RNA-seq analysis, total RNAs were subjected to cDNA synthesis and NGS library construction using the Universal Plus mRNA-Seq (NuGEN Technologies, Redwood City, CA, USA). The quality and average length of sequence library for each sample was assessed using Bioanalyser (Agilent Technologies, Santa Clara, CA, USA) and the DNA 1000 kit, respectively. The indexed samples were pooled equimolarily and sequenced on Illumina NovaSeq 6000 (150 base, paired-end reads) (Illumina, San Diego, CA, USA). The quantification of raw reads was processed using CLC Genomics Workbench v.10 software. Adaptor sequences and base with low quality or ambiguous were trimmed. The quality screened reads were mapped to Mus musculus GRCm38 genome using CLC Genomics Workbench. The mapping parameters were the following: mismatch cost 2, insertion cost 3, deletion cost 3, length fraction of 0.5 and similarity fraction of 0.8. The expression values were calculated as FPKM (Fragments Per Kilobases per Million). The differential gene expression between two or more condition was based on the fold changes of FPKM value. The genes with 2-fold change were further analysed.

### Statistical analysis

All experiments were performed from more than three independent biological preparations and each experimental value was marked as a dot in bar graphs. For real-time RT-PCR assay (Fig. 2H, I & J), the statistical analysis was performed to compare the 2^–ΔΔCt^ values of control and experimental groups from threes biological replicates. All analyses were performed using the statistical software program package Prism 5. The differences in the clinical characteristics between two groups were analysed by Chi-square test and student’s t-tests. Differences were assumed to be significant when the *p* value was <0.05.

## Results

### Changing TFAM/mtDNA modulates HNC malignancy

**Fig. 1:**
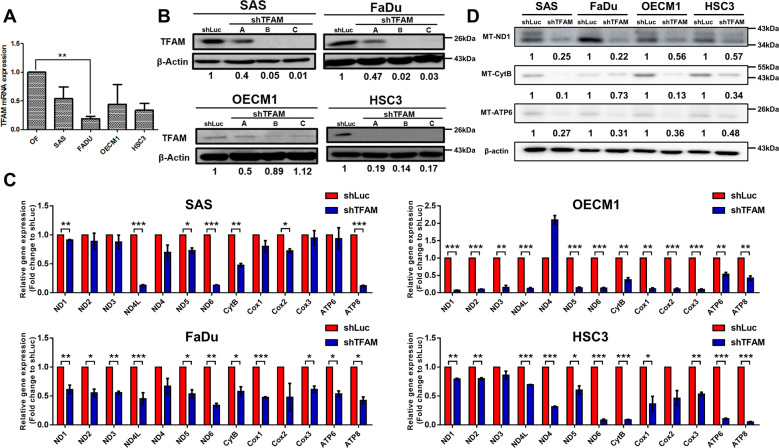
TFAM alterations lead to functional mtDNA change in HNC cells. **A** Real-time RT-PCR analysis showed decreased TFAM mRNA expression in HNC cells compared with OFs. **B** Western blot analysis showed effective changes of TFAM expression in shRNA-mediated TFAM silencing. The clone C shTFAM infected SAS/FaDu cells, clone B shTFAM infected OECM1 cells and clone A shTFAM infected HSC3 cells are used for the experiments. Functional knockdown is evident using (**C**) real-time RT-PCR and (**D**) Western blot analysis showing significant downregulation of mtDNA-encoded ETC subunit genes in TFAM silencing HNC cells. Different ETC complex genes including Complex I (ND1, ND2, ND3, ND4, ND4L, ND5, ND6), Complex III (CytB), Complex IV (E, Cox I, Cox II, Cox III) and Complex V (ATP8, ATP6) are indicated. Quantification of real-time RT-PCR analysis is represented as the fold changes of ETC gene expression in TFAM silencing (shTFAM) over control (shLuc) HNC cells. Quantification of Western blot analysis is represented as the fold changes of TFAM protein expression in TFAM silencing (shTFAM) over control (shLuc) HNC cells using Image J. Data are presented as Mean ± SEM (*N* = 3 independent biological replicates). *p < 0.05, **p < 0.01, ***p < 0.001.

To examine the roles of TFAM and its targeting mtDNA during HNC development, multiple in vitro analyses in HNC cells, with different origins in response to TFAM changes, were performed. The results showed that TFAM mRNA and protein expression is reduced in most HNC cells in contrast to normal human oral fibroblasts (OF) (Fig. 1A and Fig S1A). Next, HNC cells deficient (Fig. 1B) or enforced expressing for TFAM (Fig S1B) were utilized to better determine the role of TFAM in controlling HNC tumourigenicity. In addition to protein expression, decreased expression of mtDNA encoded ETC subunits (TFAM targets) including ND1, ND2, ND3, ND4, ND4L, ND5, ND6 (Complex I), CytB (Complex III), Cox I, Cox II, Cox III (Complex IV) as well as ATP8, ATP6 (Complex V) were detected by real-time and Western blot PCR analysis in TFAM-silenced HNC cells when compared with control cells (Fig. 1C, D). In agreement with this finding, enforced expression of TFAM also positively correlated with expression of mtDNA encoded ETC genes in TFAM overexpressing HNC cells (Fig. S1C). While no significant changes for nuclear encoded ETC proteins (NDUFB8, SDHB, UQCRC2, ATP5A) were found in TFAM knockdown HNC cells, mtDNA encoded COXII protein was greatly downregulated in TFAM-silenced SAS, OECM1 and HSC3 cells (Fig. S1D), highlighting the knockdown efficiency and specificity in our experimental setting. In short, shRNA mediated TFAM silencing could functionally reduce TFAM and its targeting cues in HNC cells.

**Fig. 2:**
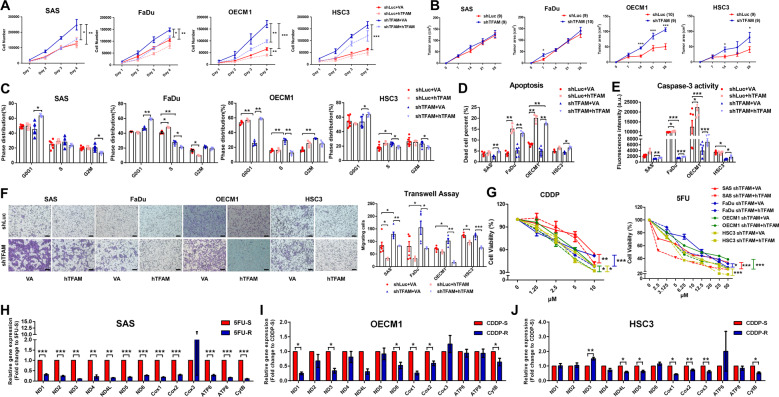
TFAM loss facilitates HNC malignancy. **A** Increased cell growth was detected in all tested TFAM-silenced HNC cells compared with control cells as TFAM overexpression (hTFAM) ameliorated this HNC cell overgrowth using Trypan exclusion assay. **B** In vivo analysis for HNC-bearing xenografic tumour growth showed that TFAM loss resulted in significant greater tumour mass compared with control tumours derived from OECM1 and HSC3 groups, but not in tumours from SAS and FaDu cells. The size at each time point in TFAM-silenced and in control HNC bearing tumours were statistically analysed. Numbers of tumour grafts were indicated in parentheses. **C** Flow cytometry based cell cycle analysis showed that TFAM loss resulted in faster cell cycling, as lower cell population in G0/G1 phase and more cells are distributed in S and G2M phases were detected in TFAM-silenced HNC cells. In contrast, hTFAM overexpression suppressed cell cycling in HNC cells, both in control and TFAM-silenced groups. By using (**D**) Annexin V based flow cytometrical analysis and (**E**) Caspase-3 activity assay, TFAM loss resulted in lower apoptotic percentage an Caspase-3 activity in HNC cells whereas enforced hTFAM expression significantly upregulated apoptotic rates and Caspase-3 activity, both in control and TFAM-silenced HNC cells. **F** Cell migration was examined using transwell assay and quantification showed that TFAM loss promoted significant increase of HNC cell migration while hTFAM expression abolished this effect, both in shLuc and TFAM-silenced HNC cells. **G** Overexpression of hTFAM sensitized TFAM-silenced HNC cells to chemotherapeutic agents CDDP and 5FU. Cell viability of TFAM-silenced HNC cells transfected with VA or hTFAM in response to 10 μM CDDP and 50 μM 5FU was statistically analysed. **H**, **I**, **J** Real-time RT-PCR analysis showed deregulated mtDNA-encoded subunit genes in 5FU-resistant (5FU-R) SAS cells and CDDP-resistant (CDDP-R) OECM1 and HSC3 cells compared with their sensitive counterparts (5FU-S and CDDP-S), respectively. Data are presented as Mean±SEM (*N* = 3 independent biological replicates). For 2 C~2 F, each dot in bar graph represents an individual biological replicate. VA Vector alone, hTFAM Full-length wild-type human TFAM. *p < 0.05, **p < 0.01, ***p < 0.001.

Next, multifaceted cellular assays were performed in TFAM knockdown and overexpressing HNC cells. By using Trypan blue exclusion assay, it was shown that TFAM knockdown led to increased HNC cell proliferation in vitro, whereas overexpression of full-length wild-type human TFAM (hTFAM) effectively downregulated cell growth in TFAM-silenced HNC cells (Fig. 2A). Similar to in vitro growth, significant larger TFAM-silenced OECM1- and HSC3-, but not SAS- and FaDu-, bearing xenografic tumours were detected in vivo (Fig. 2B and Fig. S2), implying that TFAM expression is negatively associated with HNC cell growth. This result showing differential growth behaviours between tested TFAM-silenced HNC cells could possibly attribute to distinct differences in single nucleotide variation (SNV), copy number, mRNA abundance, methylation, and tumor microenvironment (TME) profiles in different HPV^-^ HNC cells, as reported by a very recent study [30]. At mechanistic basis, further analysis found that TFAM deficiency slightly altered cell cycle phase distribution as enforced hTFAM expression significantly arrested HNC cell cycling (Fig. 2C). Moreover, by using Annexin V based apoptosis analysis and Caspase-3 activity assay, decreased apoptotic rate and reduced Caspase-3 activity was detected in TFAM-silenced HNC cells while hTFAM re-introduction predominantly triggered cellular apoptosis in both control and TFAM-silenced HNC cells (Fig. 2D, E). In sum, cell cycling and cell apoptosis both underlie TFAM mediated regulation for HNC cell growth.

As enhanced metastatic activity and drug resistance are also hallmarks of cancer cells [31, 32], cell motility and chemotherapeutic sensitivity was next assessed in HNC cells differentially expressed TFAM. By using transwell-based migration assays, TFAM loss significantly promoted HNC cell migration compared with control cells, as hTFAM overexpression largely attenuated this effect (Fig. 2F). As for the changes of therapeutic sensitivity in response to reduced TFAM expression in HNC cells, the half maximal inhibitory concentrations (IC50) of CDDP and 5FU, the most common chemotherapeutic agents for HNC in clinic, were determined. A greater IC50 for CDDP and 5FU was detected in TFAM deficient HNC cells when compared with control cells (Fig. S3); strikingly, hTFAM overexpression significantly elevated CDDP and 5FU anti-HNC efficacy in TFAM-silenced HNCs (Fig. 2G). The findings are consistent with previous studies showing that alteration of mtDNA altered chemoresistance in pancreatic cancers, suggesting that lower mtDNA may contribute resistant ability to anti-cancer drugs [33, 34]. To further elaborate the impact of mtDNA content in controlling drug resistance in HNCs, the HNC cells resistant to CDDP and 5FU as well as adjuvant photodynamic therapy (PDT) were established and mtDNA encoded ETC gene expression was analysed in these treatment-resistant HNC cells (Fig. S4, Fig. S5A, B). The results showed that, in contrast to their sensitive counterparts, most mtDNA encoded ETC gene expressions were downregulated in resistant HNC cells (Fig. 2H, I, J and Fig. S5C). These findings uncovered that mtDNA loss positively associated with chemotherapy and PDT resistance, although the underlying regulation(s) between mtDNA dysfunction and treatment resistance remain unclear. Collectively, TFAM expression negatively correlated with cell malignancy in HNCs, confirming that TFAM acts as a tumour-suppressing factor in HNCs.

### TFAM deficiency-induced Warburg phenotype in HNC cells

**Fig. 3:**
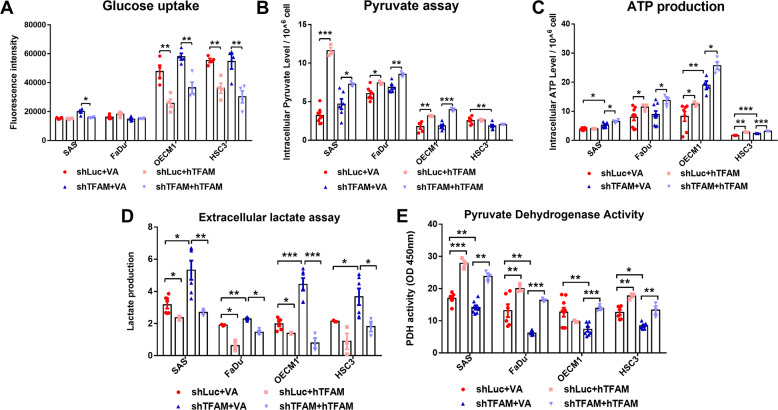
TFAM silencing elicits metabolic reprogramming in HNC cells. No significant changes of (**A**) Glucose uptake, (**B**) Intracellular pyruvate and (**C**) ATP levels were found in TFAM-silenced HNC cells compared with control cells. In contrast, hTFAM overexpression dominantly modulated these metabolic indices in both shLuc and shTFAM transfected HNC cells compared with control (VA) transfectants. **D** Significant increasing extracellular lactate levels and (**E**) decreasing Pyruvate Dehydrogenase activity were detected in most TFAM -silenced HNC cells. Introduction of hTFAM largely reversed these metabolic states in both shLuc and shTFAM transfected HNC cells compared with VA transfectants. Data are presented as Mean ± SEM. Each dot (*N* ≥ 3) in bar graph represents an individual biological replicate. VA Vector alone, hTFAM Full-length wild-type human TFAM. *p < 0.05, **p < 0.01, ***p < 0.001.

In addition to changes of intracellular signals, the metabolic plasticity was recently appreciated in numerous studies showing that cancers could evolve to adapt environmental stresses during progression [35]. Our previous findings demonstrated that manipulations for pyruvate metabolic molecules LDHA and PDHA1 led to a metabolic shift among a wide spectrum of metabolic pathways and further analysis confirmed that this metabolic reprogramming is essential for LDAH/PDHA1 mediated malignant changes in HNC cells [26]. We herein tested whether a metabolic shift could also be responsible for TFAM mediated cellular changes. Multiple bioenergetic readouts including glucose uptake, intracellular pyruvate level, extracellular lactate production, PDH activity and intracellular ATP level in TFAM-silenced HNC cells were examined. No significant difference for glucose uptake activity and intracellular pyruvate content between TFAM-silenced and control HNC cells was detected (Fig. 3A, B) whereas increasing ATP level is detected in TFAM-silenced SAS, OECM1 and HSC3 cells (Fig. 3C). Interestingly, lactate secretion was elevated (Fig. 3D) while decreased PDH activity was detected using colorimetric assay (Fig. 3E), revealing an active metabolic reprogramming in HNC cells in response to TFAM loss. Interestingly, re-introduction of hTFAM into control and TFAM-silenced HNC cells notably reverted these metabolic alterations, signifying the TFAM mediated metabolic regulations in HNC cells.

TFAM mediated metabolic shift was further evident by a LC-MS based metabolomics analysis for glycolytic and TCA cycle metabolites. The analyses shows that only metabolites in the “payoff” phase of glycolysis including 1,3 bisphosphoglycaerate, 2-/3-phosphoglycerate, and phosphoenoalpyruvate (PEP) were decreased in TFAM-silenced HNC cells while other intracellular metabolites remain unchanged (Figs. S6–7). This result could be explained as a consequence of higher glycolytic metabolism in response to TFAM loss, thereby leading to a greater consumption of glyceraldehyde-3-phosphate (G-3-P) in order to meet energetic demand. As new glucose input was not significantly altered, we further suspected that there might be alternative changes of other cellular metabolic cues, such as amino acid metabolism, that could potentially compensate deregulated mitochondrial activity in a condition of TFAM knockdown to support tumourigenic activity in HNC cells. Nevertheless, no significant changes for amino acid level were found in TFAM-silenced HNC cells when compared with control cells (Fig. S8), implying that biomolecules might be not limiting for TFAM mediated HNC oncogenic regulation.

### Akt and ERK signalling pathways regulate TFAM mediated HNC oncogenicity

**Fig. 4:**
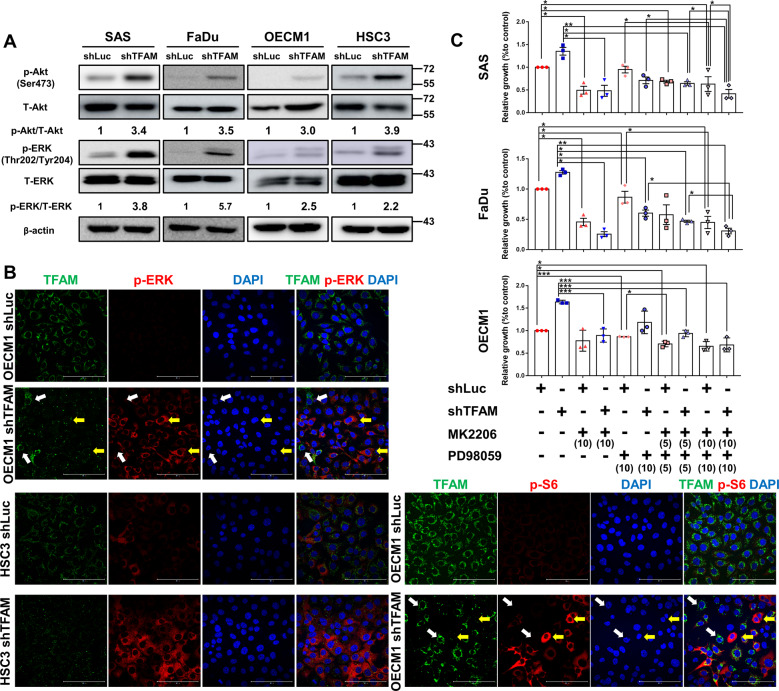
PKB/Akt and ERK signalling pathways underlie TFAM mediated malignant changes in HNC cells. **A** Western blot analysis showed increased expression of phosphorylated Akt (Ser473), phosphorylated p44/42 MAPK ERK1/2 (p-ERK, Thr202/Tyr204) and mTORC pathway effector phosphorylated S6 Ribosomal Protein (p-S6, Ser235/Ser236) in TFAM-silenced HNC cells. **B** Immunofluorescence staining analysis indicated that HNC cells with lower TFAM expression (yellow arrows) exhibited greater phosphorylated S6 (p-S6) and phosphorylated ERK (p-ERK) expression as HNC cells with higher TFAM levels displayed less p-S6 and p-ERK (white arrows). Scale bar = 100 μm. **C** Trypan blue exclusion assay for TFAM-silenced SAS, FaDu, OECM1 cells solely or co-treated with PKB/Akt inhibitor MK2206 and ERK1/2 inhibitor PD98059 showed that PKB/Akt and ERK activities contribute to TFAM-mediated increased cell growth in a dose-dependent manner. Among groups, differential cell viability was statistically compared between control and TFAM-silenced HNC cells with/without treatment of MK2206 or/and PD98059. Doses (µM) of inhibitors are shown in parentheses. Data are presented as Mean ± SEM (*N* = 3 independent biological replicates). *p < 0.05, **p < 0.01, ***p < 0.001.

While it is widely accepted that activation of various oncogenic pathways is essential for cancer development [27, 36], numerous studies have shown that Akt-mTORC and EGFR-ERK1/2 signalling pathways are highly expressed in HNC cells and crucial for HNC carcinogenic identity, both in vivo and in vitro [29, 37–40]. We therefore investigated if Akt/ERK signals are key regulators for TFAM mediated oncogenic changes in HNC cells. Western blot analysis showed that phosphorylated Akt (Ser473) and phosphorylated p44/42 MAPK ERK1/2 (Thr202/Tyr204) pathway were all upregulated in TFAM-silenced HNC cells compared with control cells (Fig. 4A) while enforced hTFAM expression abolished TFAM-silenced mediated Akt/ERK hyperactivity (Fig. S9), suggesting that TFAM might act as one of the upstream regulators in controlling Akt/ERK signalling activity. At the single-cell level, IFA further confirmed that HNC cells, with reduced TFAM expression, expressed a higher amount of phosphorylated Akt/ERK/S6 proteins, indicating that TFAM mediated malignant changes could be made through the modulations of ERK1/2 and Akt-mTORC-S6 signalling pathways (Fig. 4B). To further define the role of Akt/ERK signalling pathway in regulating TFAM-mediated neoplastic characteristics, PKB/Akt inhibitor MK2206 and ERK1/2 inhibitor PD98059 were applied in TFAM-silenced HNC cells and cell proliferation was examined. The results showed that efficient inhibition of Akt and ERK1/2 activity (Fig. S10) significantly abolished increased cell growth in TFAM-silenced HNC cells. Strikingly, a combinational treatment of MK2206 and PD98059 exhibited a dose-dependent synergetic effect in controlling TFAM mediated HNC cell growth (Fig. 4C). These results confirmed a novel notion that mtDNA loss in response to TFAM knockdown could trigger cytosolic signalling alteration. Interestingly, previous studies have reported that the recruitment of Akt protein to mitochondria could inactivate PDC thus resulting in downregulation of Oxidative Phosphorylation (OxPhos) pathway under hypoxic condition [41], further supporting a potential crosstalk between Akt signal and mitochondrial metabolism. Collectively, these findings provided an alternative scheme for development of TFAM/Akt-ERK combinational anti-cancer therapeutic strategy for HNCs.

### TFAM loss regulates HNC cell growth via elevated cellular oxidative stress

**Fig. 5:**
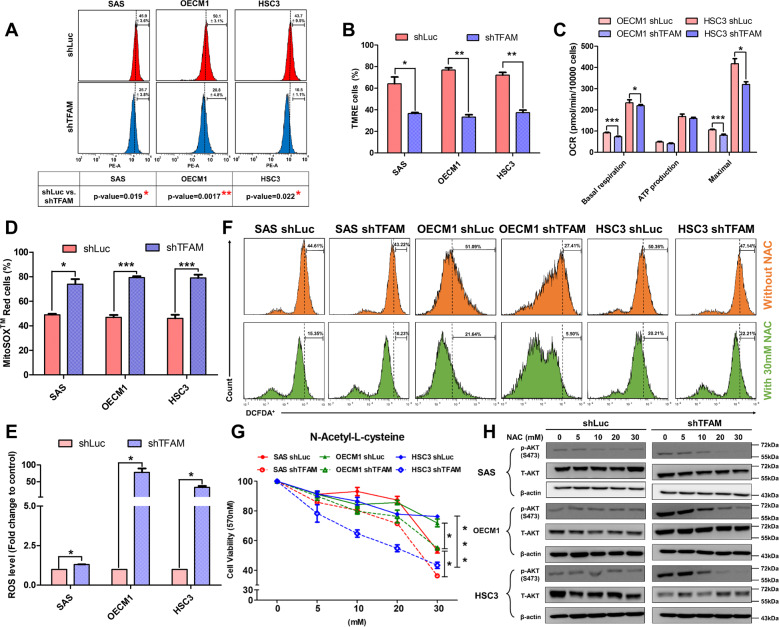
TFAM loss affects mitochondrial physiology in HNC cells. **A** Flow cytometrical analysis showed that TFAM loss led to decreased mitochondrial activity by Mitotracker Red staining in HNC cells. **B** Flow cytometrical analysis showed that TFAM loss led to decreased mitochondrial membrane potential by using TMRE staining in HNC cells. **C** Seahorse analysis indicated a drop of basal respiration, maximal respiration and ATP production in TFAM-silenced HNC cells. A significant increased cellular and mitochondrial-derived ROS content was detected using (**D**) MitoSOX staining and (**E**) a flow cytometry based DCFDA assay in TFAM-silenced HNC cells compared with their control counterparts. **F** ROS content was detected by flow cytometry in SAS, OECM1 and HSC3 cells with 30 mM NAC treatment. **G** MTT assay showed a greater drop of cell viability in TFAM-silenced HNC cells compared with control cells in response to NAC treatment. Cell viability of control and TFAM-silenced HNC cells treated with 30 mM NAC was statistically analysed. **H** Western blot analysis for total and phosphorylated Akt in SAS, OECM1 and HSC3 cells treated with different NAC concentrations. Data are presented as Mean ± SEM (*N* = 3 independent biological replicates). *p < 0.05, **p < 0.01, ***p < 0.001.

As TFAM loss could activate metabolic reprogramming in HNC cells, the mitochondrial physiological alteration(s) were further emphasised in TFAM-silenced HNC cells. Mitotracker red (Fig. 5A) and Tetramethylrhodamine, ethyl ester (TMRE) (Fig. 5B) staining analyses revealed that mitochondrial structure and activity were both decreased in TFAM-silenced HNC cells compared with control cells. Furthermore, by using Seahorse Mito-stress analyser, it was found that mitochondrial basal respiration, mito-ATP and maximal respiration were downregulated in response to TFAM reduction in HNC cells compared with control cells (Fig. 5C). Surprisingly, both mitochondrial derived ROS determined by MitoSOX Red assay (Fig. 5D) and cellular Reactive Oxygen Species (ROS) detected by H2DCFDA assay (Fig. 5E), were upregulated in TFAM-silenced HNC cells compared with control counterparts. To better examine whether the increasing ROS is responsible for HNC malignancy, cell growth change in TFAM-silenced HNC cells incubated with N-acetylcysteine (NAC), a well-known antioxidant compound [42], was measured (Fig. 5F). It was interestingly found that a rapid decrease of HNC cell viability was found in TFAM-silenced HNC cells compared with control group (Fig. 5G). This ROS mediated regulation for cell growth could be possibly through oncogenic signalling cues since these Akt proteins were downregulated in a larger scale in TFAM-silenced HNC cells than control cells treated with NAC (Fig. 5H). In short, our data underpin a possibility that TFAM loss might trigger ETC dysfunction thereby resulting in greater ROS leak and eventually increased oncogenicity. Taken together, our results demonstrated that TFAM loss facilitated HNC cell malignancy likely via intrinsic metabolic reprogramming away from mitochondrial metabolism towards aerobic glycolysis without altering external nutrition uptake and nitrogen metabolic pathway and upregulated ROS/Akt/ERK regulatory pathway.

### Decreased TFAM and its downstream genes in human HNCs

**Fig. 6:**
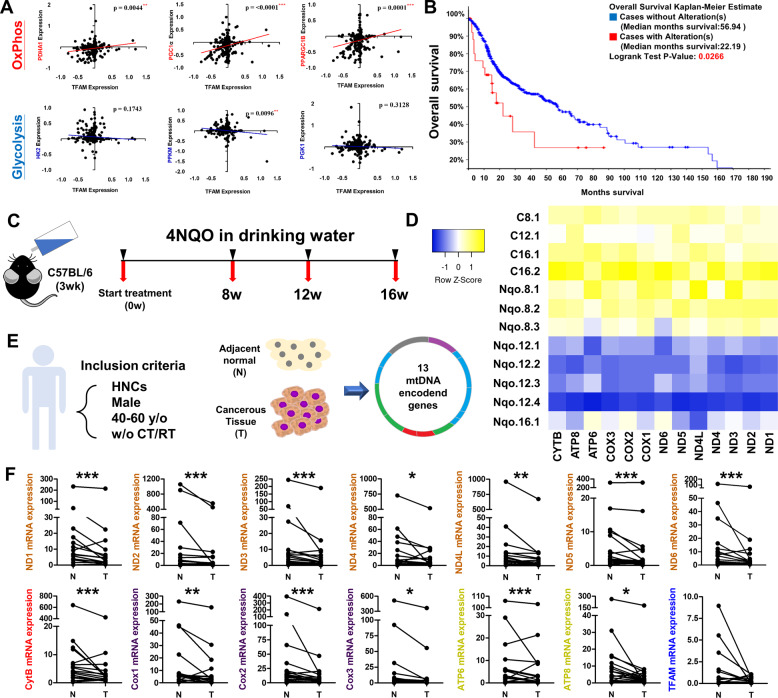
Clinical Impacts of TFAM and mtDNA-encoded ETC in HNCs. **A** Mitochondrial factor (PDHA1, PGC1α, PPARGC1β) were detected in TCGA based database; whereas negative correlations between TFAM and glycolytic enzymes (HK2, PFKM, PGK1) were found in HNCs using The Cancer Genome Atlas (TCGA) database (*N* = 273). **B** Kaplan–Meier analysis for overall survival rates in HNC patients classified by TFAM genetic alteration using TCGA database (*N* = 519). HNC cases with TFAM K141N mutational alteration have significant poorer overall survival (*N* = 26, median months’ survival: 22.19) than HNC cases without TFAM alteration(s) (*N* = 462, median months survival: 56.94). **C** Experimental scheme of Nqo mediated induction for mouse tongue cancer. **D** Heatmap of mtDNA encoded ETC gene expression in control group (C; *N* = 4) and 4NQO treatment group (Nqo; *N* = 8). Rows represent control and Nqo treatment group; column represent 13 mtDNA encoded ETC genes. For different group, C/Nqo.X.Y is used where X indicates treatment time (weeks) and Y represents mouse number. The color scale runs from blue (low intensity) to white (medium intensity), to yellow (strong intensity) calculated by z-score. **E**, **F** Analysis for mtDNA encoded ETC genes in cancerous tissues (T) and their corresponding adjacent normal tissues (*N*) from HNC patients (*N* = 18) with indicated criteria was performed using real-time RT-PCR analysis. Significant decreased mtDNA encoded ETC and TFAM mRNA expression in clinical tumourous tissues compared with their normal counterparts was detected. *p < 0.05, **p < 0.01, ***p < 0.001.

As previous studies found that TFAM and mtDNA levels differentially correlated with colorectal and liver cancer prevalence [43, 44], the association between TFAM expression and HNC oncogenicity needs further determination. To this end, the expression of TFAM and its target genes in HNC and their corresponding normal tissues was analysed using The Cancer Genome Atlas (TCGA) based databases. The results showed that TFAM mRNA level was positively correlated with mRNA expression for OxPhos factors (PDHA1, PGC1α, PPARGC1β) but negatively associated with glycolytic enzymes (HK2, PFKM, PGK1) (Fig. 6A). Interestingly, HNC patients with TFAM genetic mutation (K141N) exhibited worse Overall Survival (OS) rate than HNC patients without TFAM alteration (Median Survival: 22.19 months vs. 56.94 months) indicating that maintenance of TFAM integrity could be essential for better prognosis in HNC patients (Fig. 6B). In order to clarify whether mtDNA drop could also be evident during HNC progression in vivo and in clinic, 13 mtDNA encoded ETC genes from 8-, 12- and 16-week control and Nqo-induced mouse tongue tissues was examined. By RNA-seq analysis, it was found that mtDNA encoded ETC subunits were transcriptionally downregulated in Nqo groups compared to control group and, between Nqo treated groups, mtDNA encoded ETC genes were dropped with prolonged Nqo treatment, suggesting that decrease of mtDNA encoded ETC subunits could be a predisposition factor for mouse tongue cancer development (Fig. 6C, D).

The expression of TFAM and its downstream targets was further examined in paired adjacent normal (N) and tumour (T) clinical tissues from HNC patients (*N* = 18; Table S1). In agreement with database analysis, TFAM mRNA expression of all mtDNA encoded ETC genes were significantly downregulated in tumours compared with corresponding normal tissues, suggesting that decreased TFAM levels might be important for HNC development (Fig. 6E, F). Further analysis to define a potential association of TFAM/mtDNA encoded ETC gene expression and disease progression stratified by TNM scaling was next determined. Even though the data showed no statistical significance, all mtDNA encoded ETC genes showed a decreasing trend over a T (from T1 to T4) and N stages (from N0 to N2) (Fig. S11–12), suggesting that mtDNA encoded ETC genes reversely associated with disease severity. To draw a more definite conclusion, a larger number HNC samples would be required.

## Discussion

In the present study, we discovered that lower TFAM and mtDNA expression led to decreased mitochondrial activity, which elicited oncogenic pathways to promote tumour proliferation, mobility and therapeutic sensitivity. At molecular basis, we further demonstrated that ERK1/2 and Akt-mTORC-S6 pathways were highly active in TFAM-silenced HNC cells and treatment of ERK/Akt inhibitors effectively repealed TFAM-mediated malignant changes. In contrast, enforced TFAM expression suppressed cancerous identity and reversed Warburg phenotype, indicating that TFAM actively contribute to modulation of HNC malignant features. In agreement with in vitro and in vivo findings, it was also found that TFAM and mtDNA encoded ETC genes were downregulated in Nqo treated mouse tongue cancer and clinic HNC tissues compared with their normal counterparts. In summary, our findings provide strong evidence showing that [1] TFAM is critical for mtDNA replication/transcription in HNCs [2]; TFAM loss/overexpression is sufficient to modulate HNC malignancy via metabolic reprogramming towards a cancer favourable metabolic state and modulations of oncogenic effector Akt and ERK pathways; and [3] expression of TFAM and mtDNA encoded ETC genes could predict HNC progression in preclinical and clinical specimens prior to conventional treatments.

Based on previous investigations, TFAM does not always act as a suppressor in cancers, as summarized in Table S5, while TFAM is enriched and could promote cancer cell proliferation and metastasis in bladder [45], oesophageal [46], gastric [47] and colon cancers [48]. It is suspected that the discrepant roles of TFAM in different cancers could possibly be attributed to distinct metabolic features in order to adapt to different tumour microenvironments [49]. Moreover, as genetic mutations/polymorphisms of mtDNA genes might also be a key factor in controlling tumour cell malignancy [50, 51]; a detection of mutations in mt-ND2, mt-ND4, mt-ATP8 and mt-CytB in tested HNC cell lines (Table S6) highlighted great necessity to further define the impacts of these mtDNA mutations during HNC development, in an attempt to find a diagnostic marker for HNCs.

Another interesting discoveries from our results are [1]: TFAM seems impacted differentially for extrinsic and intrinsic metabolic cues since no significant changes for glucose uptake, intracellular ATP level and pyruvate content were detected whereas elevated aerobic glycolysis was evident in TFAM-silenced HNC cells. Results from these metabolic assays suggested that TFAM loss does alter HNC cell metabolism; however, the reprogramming seems to be the outcome of rewiring of intracellular metabolites but not external nutrient inputs and [2] Unlike the dogmatic role of ROS to trigger cell death, current findings surprisingly demonstrated that ROS level is elevated in mitochondrial defective TFAM-silenced HNC cells and increased ROS level likely plays a critical role in regulating tumour growth through the activation of pro-tumourgenic cues. In agreement with our data, numerous studies have also found that ROS could induce PI3K/Akt/mTORs activity in various cancers [52, 53], suggesting that excessive ROS may serve as a promoter for neoplastic transformation. Taken together, TFAM expression is critical to control metabolic mediated regulations in HNCs.

It is widely accepted that cancers are metabolically dynamic in order to survive in different environmental challenges. It therefore becomes convincing that targeting tumour metabolic plasticity could be capable of improving therapeutic efficacy of anti-cancer schemes [53]. In agreement with this concept, we found that suppression of TFAM-silenced induced ERK1/2 and Akt activity by commercial inhibitors could effectively abolish cell viability in highly-proliferating TFAM-silenced HNC cells, implying that targeting intracellular molecular cues, in combination with metabolic manipulation, could likely develop a more efficient treatment regimen in the clinic. In addition to intracellular molecular changes, mitochondrial manipulation could also result in mitochondria independent metabolic alterations in different cancers. By taking advantage of transcriptomics technology, a previous study found that TFAM loss led to increased angiogenesis and invasion as well as genes relates to amino acid metabolism [e.g., SLC1A5 (ASCT2) and SLC1A4 (ASCT1)] in melanoma cells [49], suggesting that, in order to gain better anti-cancer therapeutic efficacy, it is important to define molecular and metabolic features upon mitochondrial manipulations in order to target the correct cue(s) for combinational therapy.

**Fig. 7:**
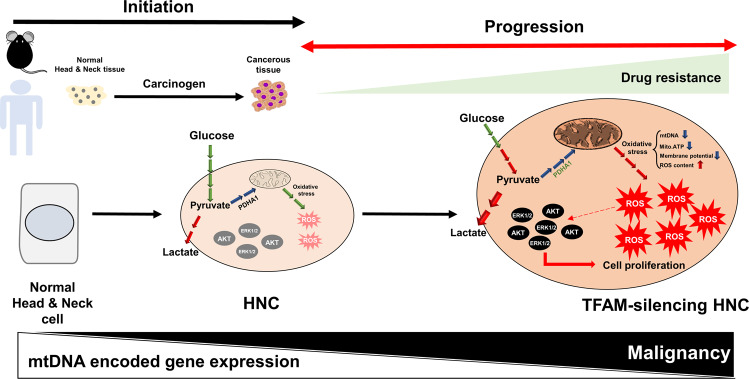
Decreasing TFAM/mtDNA contributes HNC pathogenesis. mtDNA encoded gene expression declines during HNC initiation. Further loss of TFAM/mtDNA triggers HNC malignant progression via multifaceted metabolic and molecular alterations.

In conclusion, we highlighted the prominent role of TFAM in regulating HNC malignancy and confirmed that TFAM could be a key tumour suppressor during HNC tumourigenesis (Fig. 7). We hope our findings could potentially shed the light on the development of new TFAM/mtDNA based diagnostic scheme to early detection of HNCs.

## Supplementary information

Supplementary figure legend

Supplementary Fig.1

Supplementary Fig.1 (Continue.1)

Supplementary Fig.1 (Continue.2)

Supplementary Fig.2

Supplementary Fig.3

Supplementary Fig.4

Supplementary Fig.5

Supplementary Fig.5 (Continue)

Supplementary Fig.6

Supplementary Fig.7

Supplementary Fig.8

Supplementary Fig.9

Supplementary Fig.9 (Continue)

Supplementary Fig.10

Supplementary Fig.11

Supplementary Fig.12

Supplementary Table 1-6

## Data Availability

The data presented in the current study are available upon reasonable request from the corresponding author.
