# Supplementary material for: Mitochondrial genome and its regulator TFAM modulates head and neck tumourigenesis through intracellular metabolic reprogramming and activation of oncogenic effectors
Source: Cell Death Dis. 2021 Oct 18;12(11):961. doi: 10.1038/s41419-021-04255-w (PMC8523524; doi:10.1038/s41419-021-04255-w)
Supplement: Supplementary file 1 — Supplementary figure legend [file 41419_2021_4255_MOESM1_ESM.docx]

**Supplementary Figure legends**

**Supplementary Fig. 1 Establishment of TFAM overexpressing HNC cells**

(A) Western blot analysis showed TFAM protein expression in normal oral fibroblasts and HNC cells. (B) Western blot analysis showed effective TFAM overexpressing in HNC cells. Myc sequence was fused with TFAM gene to form TFAM-Myc protein. (C) mtDNA encoded genes were upregulated in TFAM overexpressing HNC cells. (D) Western blot analysis showed differential expression of respiratory proteins in TFAM silencing HNC cells. Data are presented as Mean±SEM (N=3 independent biological replicates). *p<0.05, **p<0.01.

**Supplementary Fig. 2 *In vivo* analysis in response TFAM loss**

*In vivo* analysis for HNC-bearing xenografic tumour growth showed that TFAM silencing resulted in greater tumour weight.

**Supplementary Fig. 3 TFAM loss enhanced chemoresistance.**

Upregulation of half maximal inhibitory concentration (IC50) for chemotherapeutic agent CDDP and 5FU was detected in response to TFAM loss in HNC cells. *p<0.05, **p<0.01, ***p<0.001

**Supplementary Fig. 4 Establishment of CDDP and 5FU resistance HNC cells**

Greater cell viability was detected in CDDP resistant (A) OECM1 and (B) HSC3 cells and in (C) 5FU resistance SAS cells. *p<0.05, **p<0.01, ***p<0.001

**Supplementary Fig. 5 mtDNA encoded ETC genes were altered in PDT resistant SAS and CA 9-22 cells.**

Higher cell viability was shown in PDT resistance (A) SAS and (B) CA 9-22 cells. Lower mtDNA encoded ETC genes was detected in PDT resistance (C) SAS and (D) CA 9-22 cells compared with its sensitive counterpart. Data are presented as Mean±SEM (N=3 independent biological replicates). *p<0.05, **p<0.01, ***p<0.001.

**Supplementary Fig 6. Glycolytic metabolites in TFAM-silenced HNC cells by LC-GC mass analysis**

(A) Schematic illustration of glycolysis. Quantification of (B) Glucose, (C) Fructose-1,6-Bisphosphate, (D) Glucose 6-phosphate/ Fructose 6-phosphate, (E) 1,3-diphosphoglycerate, (F) 3-phosphoglycerate/2-phosphoglycerate, (G) Phosphoenolpyruvate in TFAM-silenced OECM1 and HSC3 cells. Data are presented as Mean±SEM (N=3 independent biological replicates).

**Supplementary Fig 7 Tricarboxylic acid cycle (TCA) metabolites in TFAM-silencing HNC cells by LC-GC mass analysis**

(A) Schematic illustration of TCA cycle. Quantification of (B) Citrate/Isocitrate, (C) Fumarate and (D) Malate in TFAM-silenced OECM1 and HSC3 cells. Data are presented as Mean±SEM (N>=3).

**Supplementary Fig 8. Amino acid content in TFAM-silencing HNC cells by LC-GC mass analysis**

Quantification of (A) Threonine, (B) Lysine, (C) Histidine, (D) α-Alanine, (E) Aspartate, (F) Glutamic Acid, (G) Proline, (H) Arginine, (I) Valine, (J) Glutamic Acid, (K) Methionine, (L) Phenylalanine, (M) Tryptophan in TFAM-silenced OECM1 and HSC3 cells.

**Supplementary Fig 9. TFAM overexpression abolished Akt and ERK1/2 activity in HNC cells**

Western blot analysis for total and phosphorylated ERK, Akt and TFAM expression in shLuc and TFAM silencing HNC cells transfected with full-length wild-type human TFAM gene. Quantification was shown as fold changes of Akt/ERK activity in cells transfected with hTFAM over control group.

**Supplementary Fig 10. Detection for treatment effect of MK2206 and PD98059**

Western blot analysis for total and phosphorylated (A) Akt and (B) ERK1/2 in SAS, FaDu, OECM1 cells treated with MK2206 and PD98059.

**Supplementary Fig 11. Association of TFAM/mtDNA encoded ETC gene expression and disease progression stratified by tumor stage in HNC patients**

Comparison of mtDNA encoded ETC genes (A) ND1, (B) ND2, (C) ND3, (D) ND4, (E) ND4L, (F) ND5, (G) ND6, (H) Cox1, (I) Cox2, (J) Cox3, (K) ATP6, (L) ATP8, (M) CytB and (N) TFAM. in tumour tissues from HNC patients stratified by tumor stages by real-time PCR analysis.

**Supplementary Fig 12. Association of TFAM/mtDNA encoded ETC gene expression and disease progression stratified by tumor node status in HNC patients**

Comparison of mtDNA encoded ETC genes (A) ND1, (B) ND2, (C) ND3, (D) ND4, (E) ND4L, (F) ND5, (G) ND6, (H) Cox1, (I) Cox2, (J) Cox3, (K) ATP6, (L) ATP8, (M) CytB and (N) TFAM. in tumour tissues from HNC patients stratified by tumor node status by real-time PCR analysis.
