# Supplementary figures and images for: Mitochondrial genome and its regulator TFAM modulates head and neck tumourigenesis through intracellular metabolic reprogramming and activation of oncogenic effectors
Source: Cell Death Dis. 2021 Oct 18;12(11):961. doi: 10.1038/s41419-021-04255-w (PMC8523524; doi:10.1038/s41419-021-04255-w)

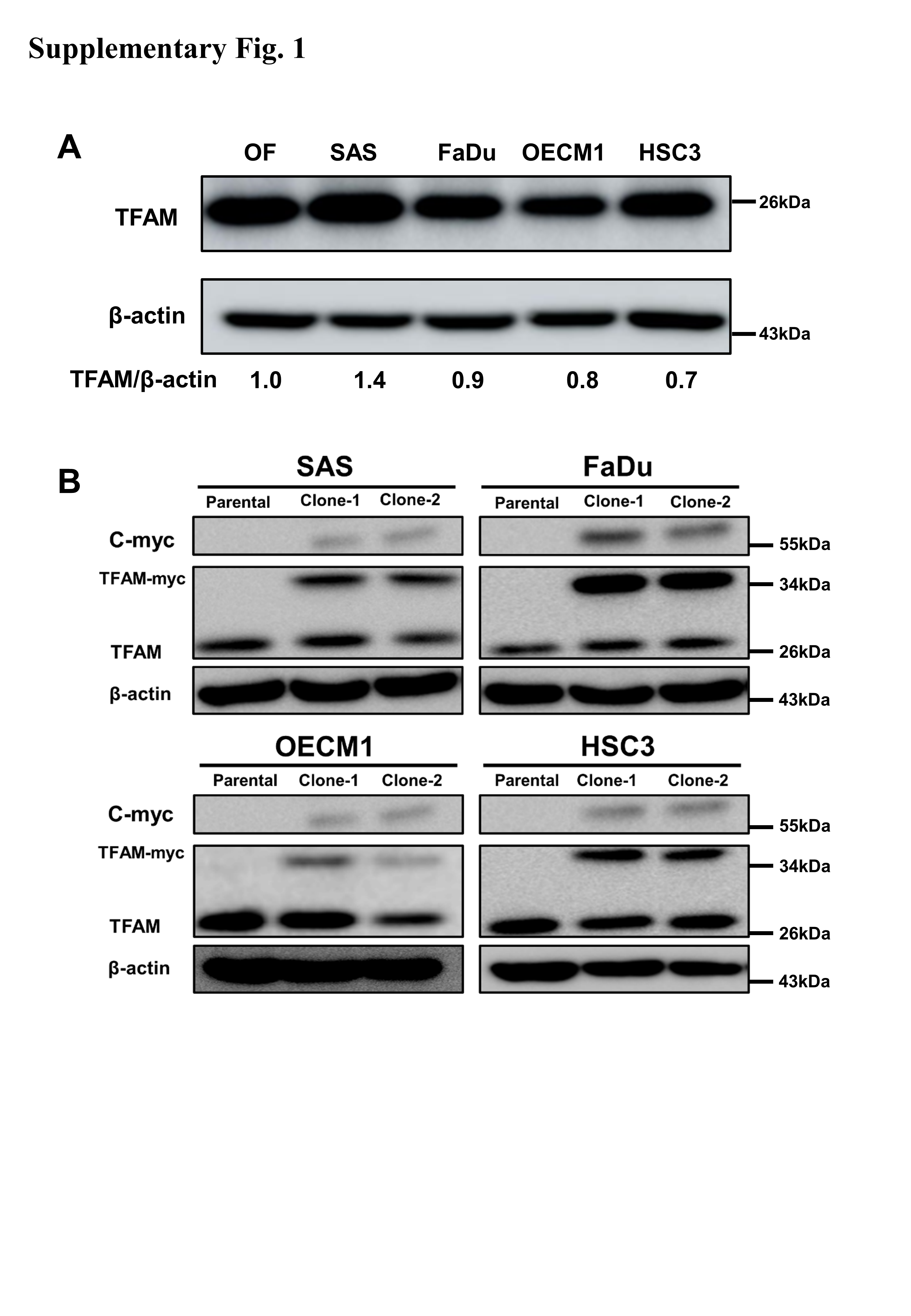

Supplement: Supplementary file 2 — Supplementary Fig.1 [file 41419_2021_4255_MOESM2_ESM.tif]

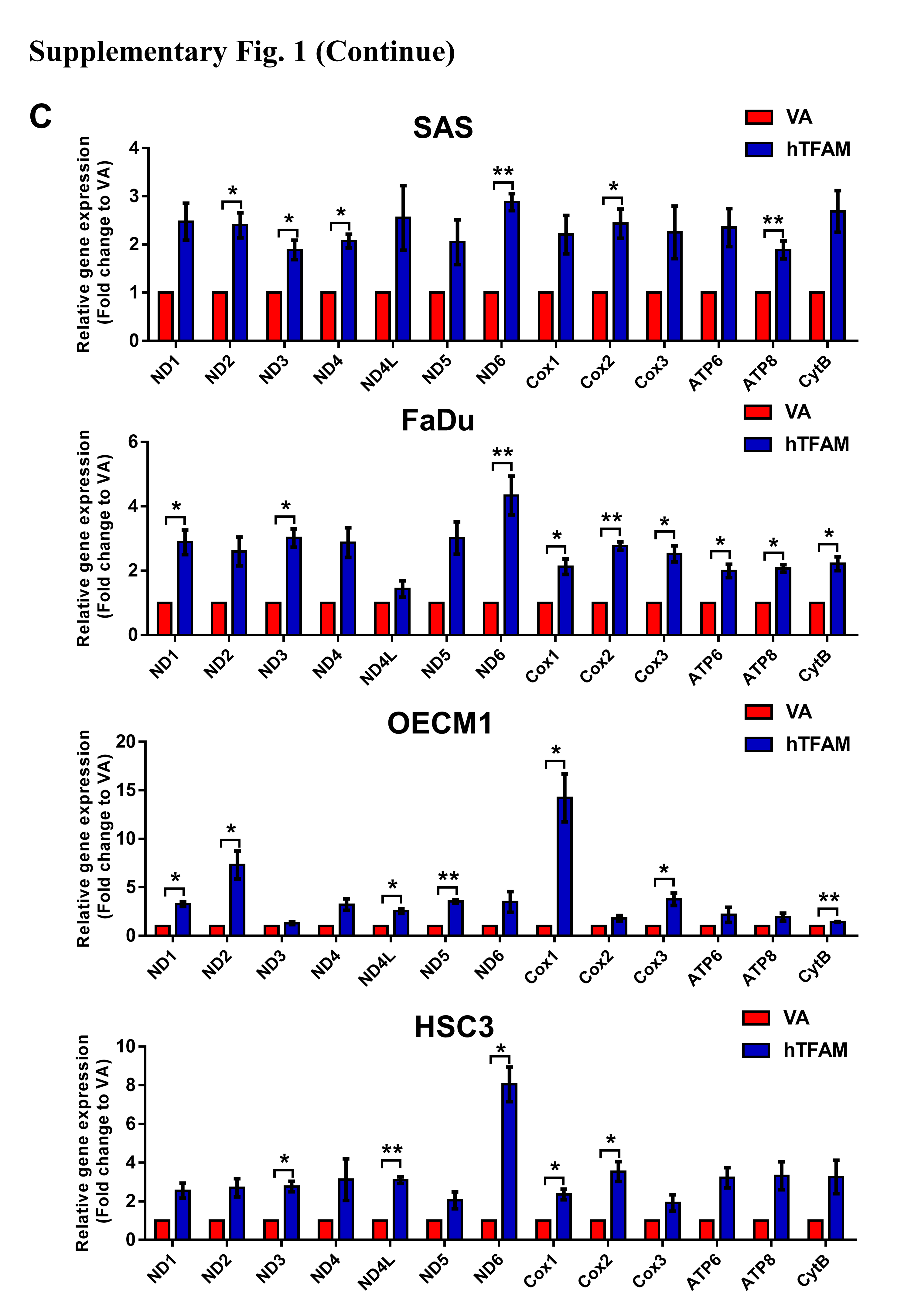

Supplement: Supplementary file 3 — Supplementary Fig.1 (Continue.1) [file 41419_2021_4255_MOESM3_ESM.tif]

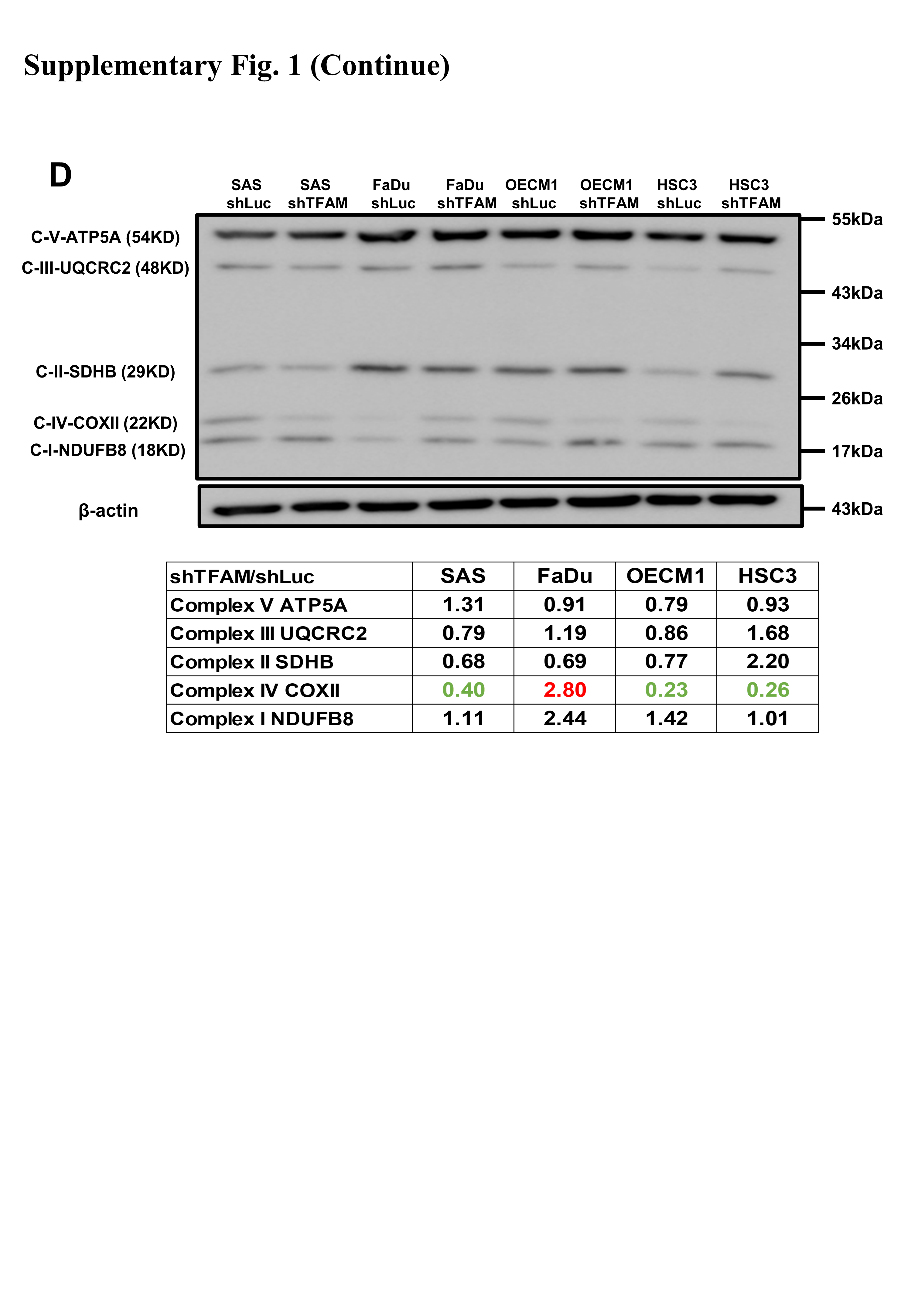

Supplement: Supplementary file 4 — Supplementary Fig.1 (Continue.2) [file 41419_2021_4255_MOESM4_ESM.tif]

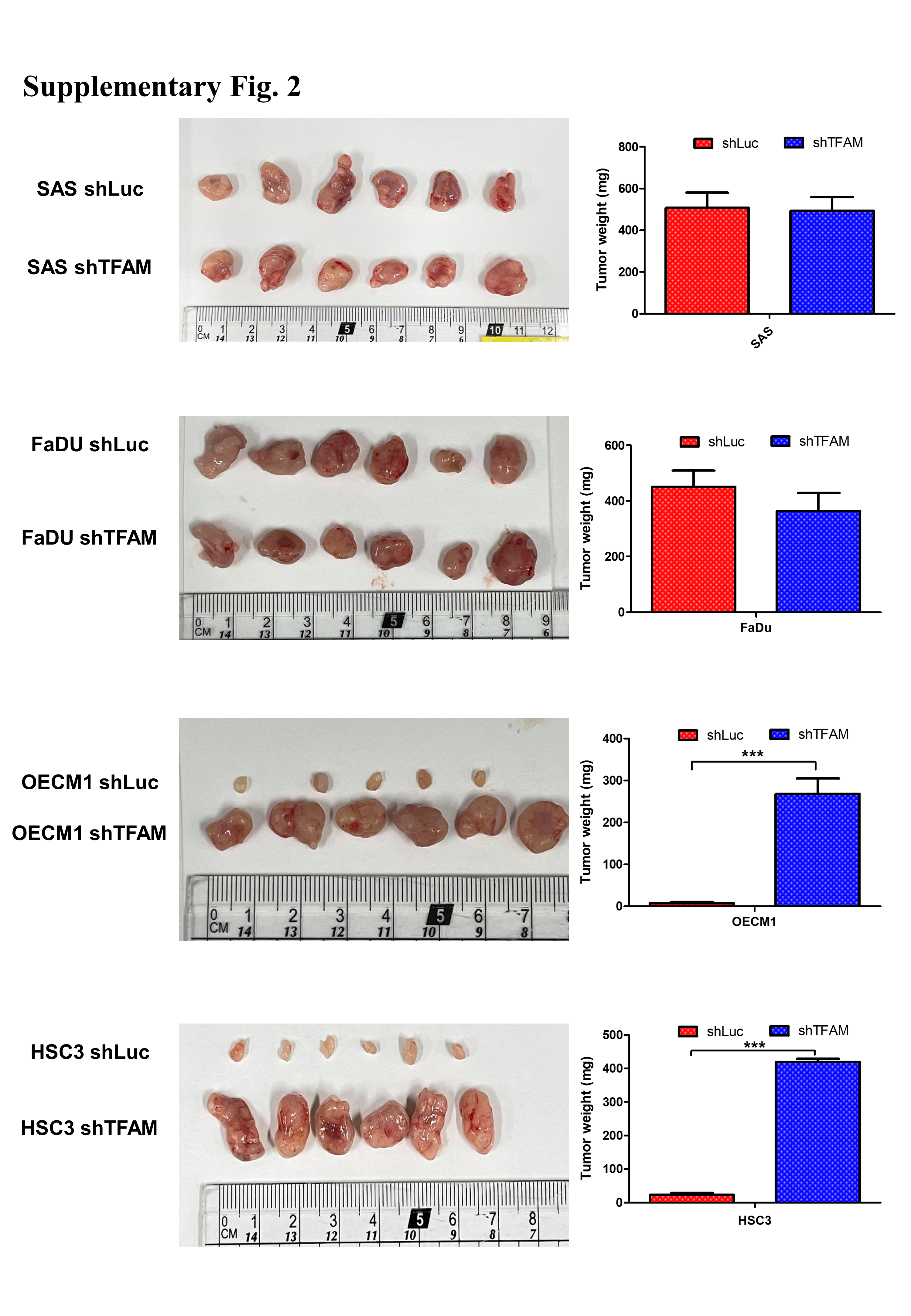

Supplement: Supplementary file 5 — Supplementary Fig.2 [file 41419_2021_4255_MOESM5_ESM.tif]

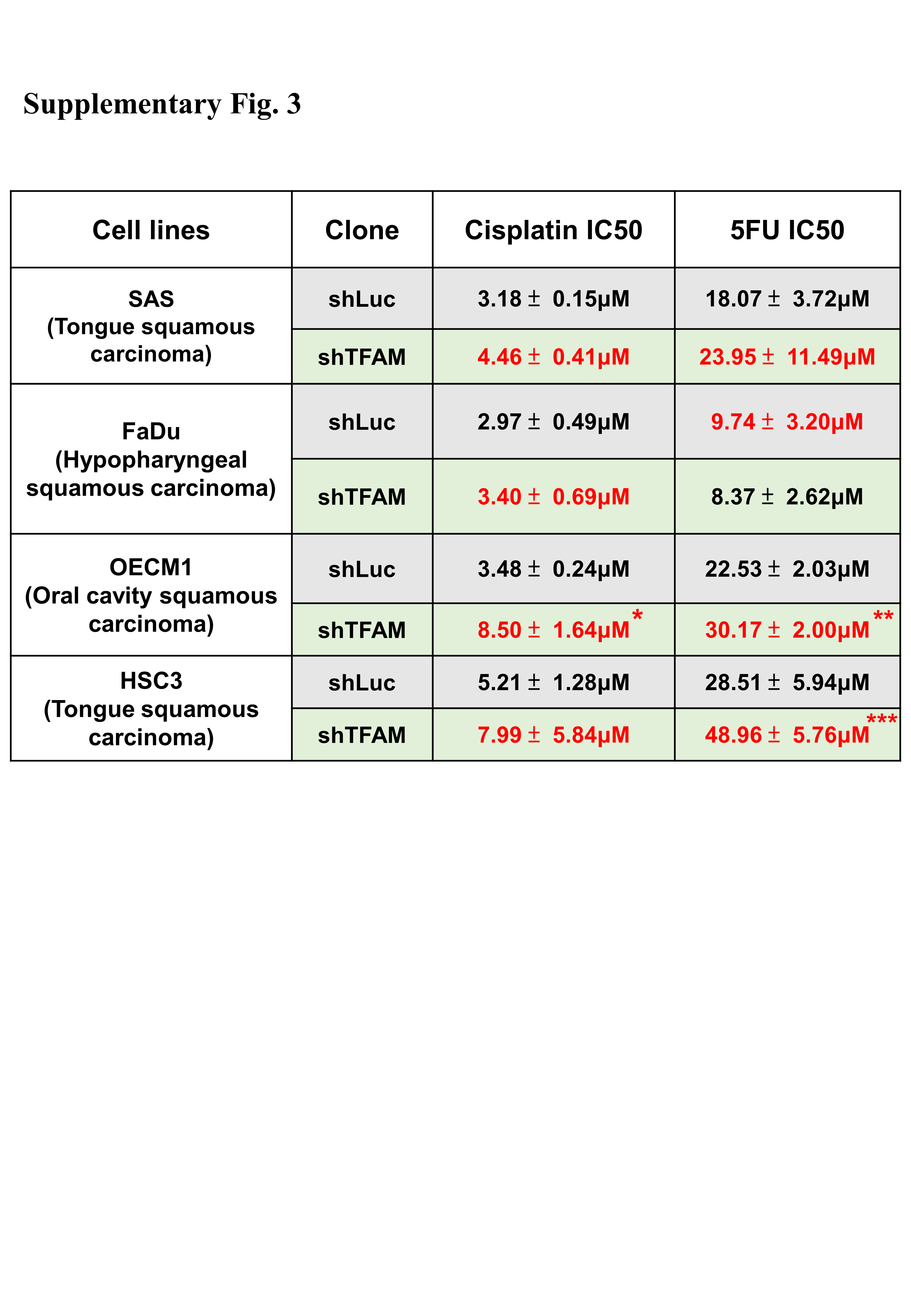

Supplement: Supplementary file 6 — Supplementary Fig.3 [file 41419_2021_4255_MOESM6_ESM.tif]

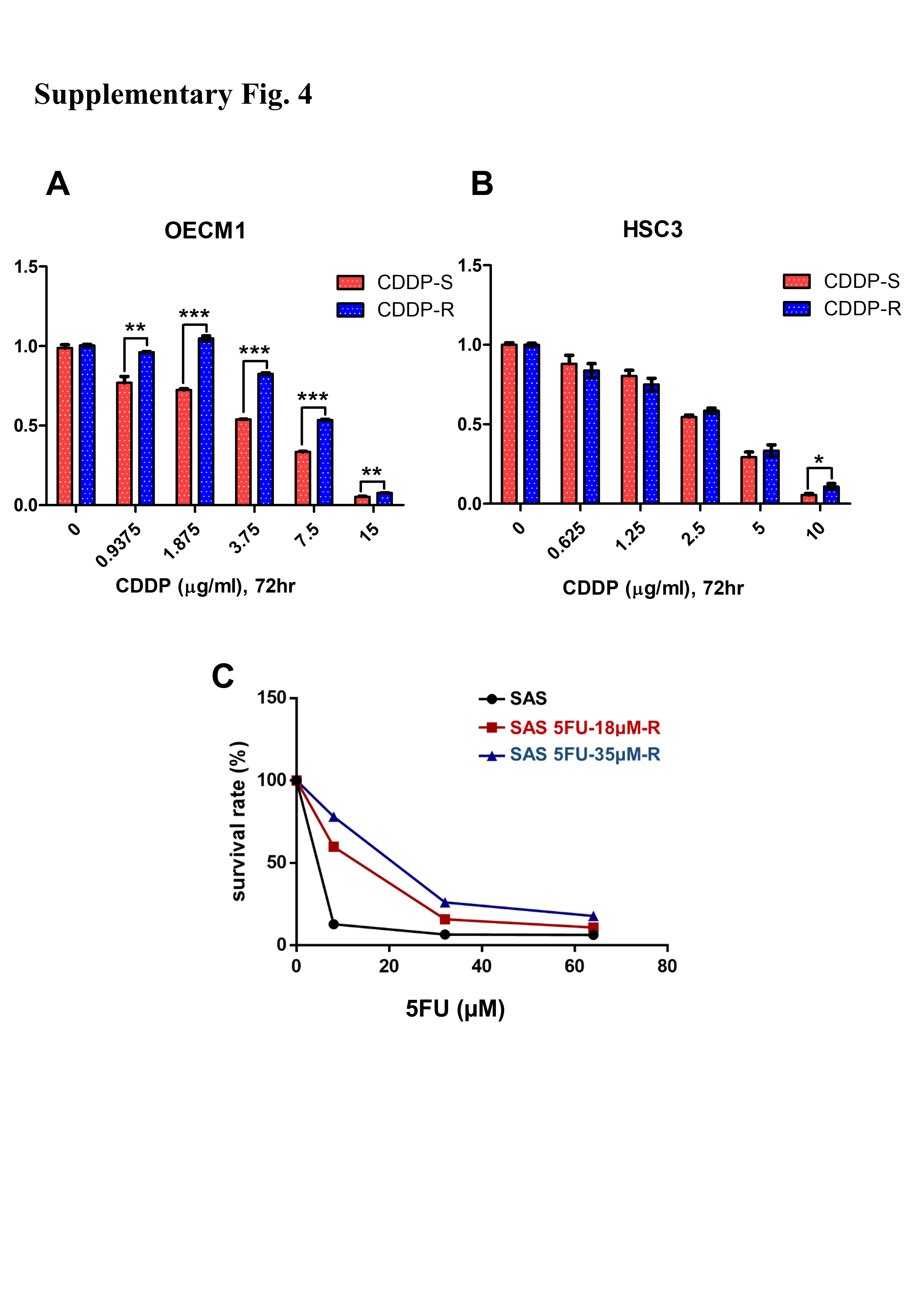

Supplement: Supplementary file 7 — Supplementary Fig.4 [file 41419_2021_4255_MOESM7_ESM.tif]

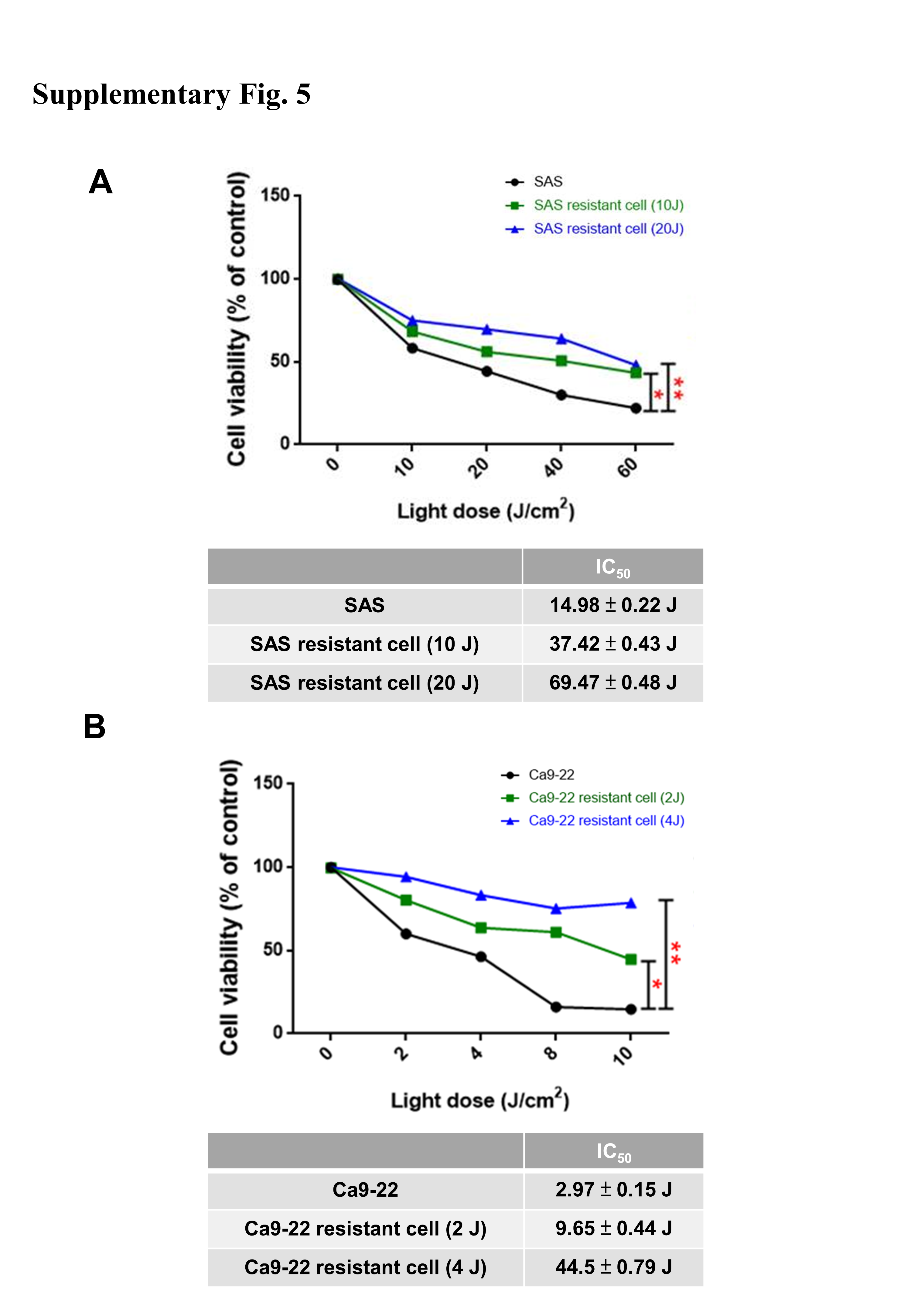

Supplement: Supplementary file 8 — Supplementary Fig.5 [file 41419_2021_4255_MOESM8_ESM.tif]

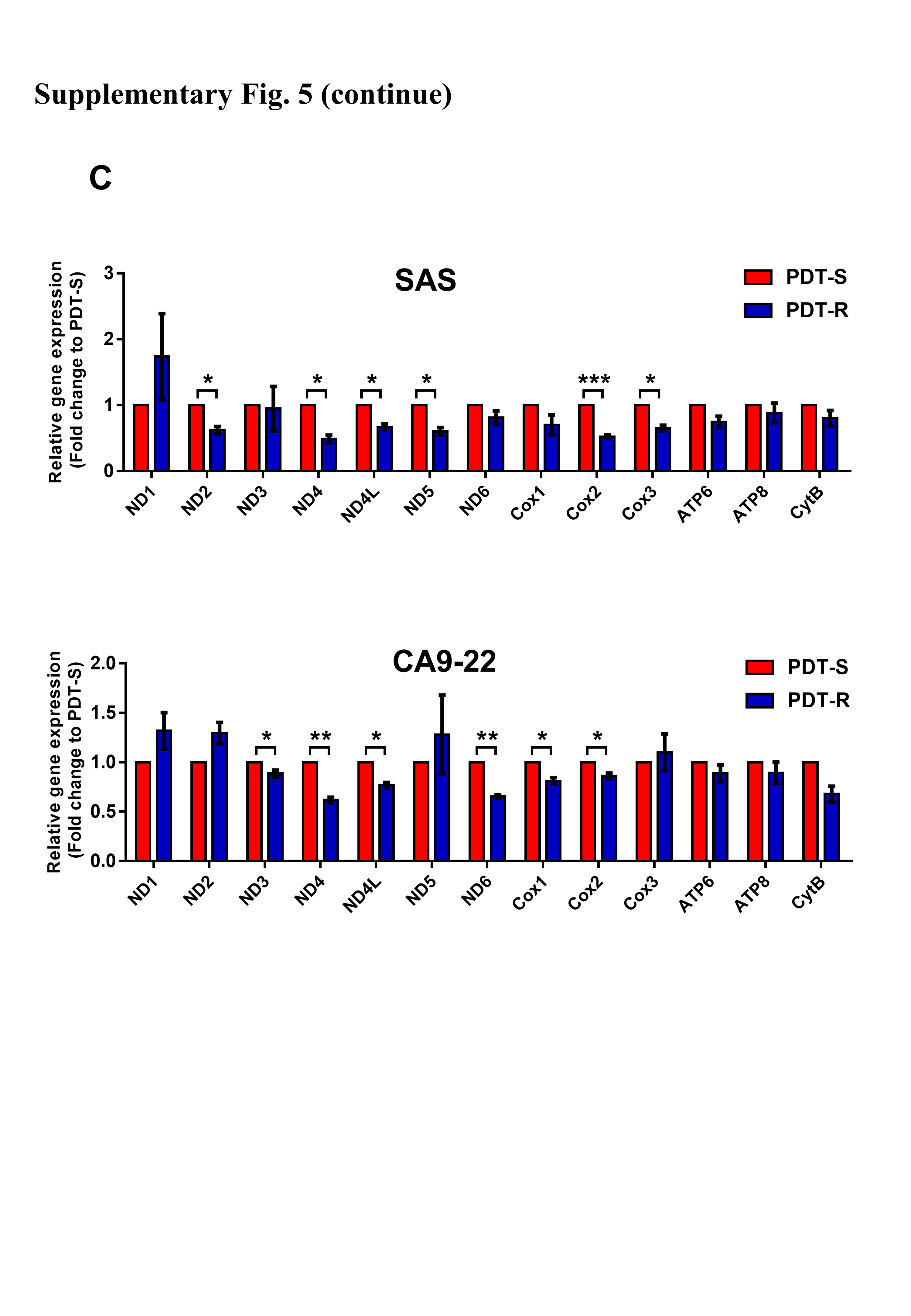

Supplement: Supplementary file 9 — Supplementary Fig.5 (Continue) [file 41419_2021_4255_MOESM9_ESM.tif]

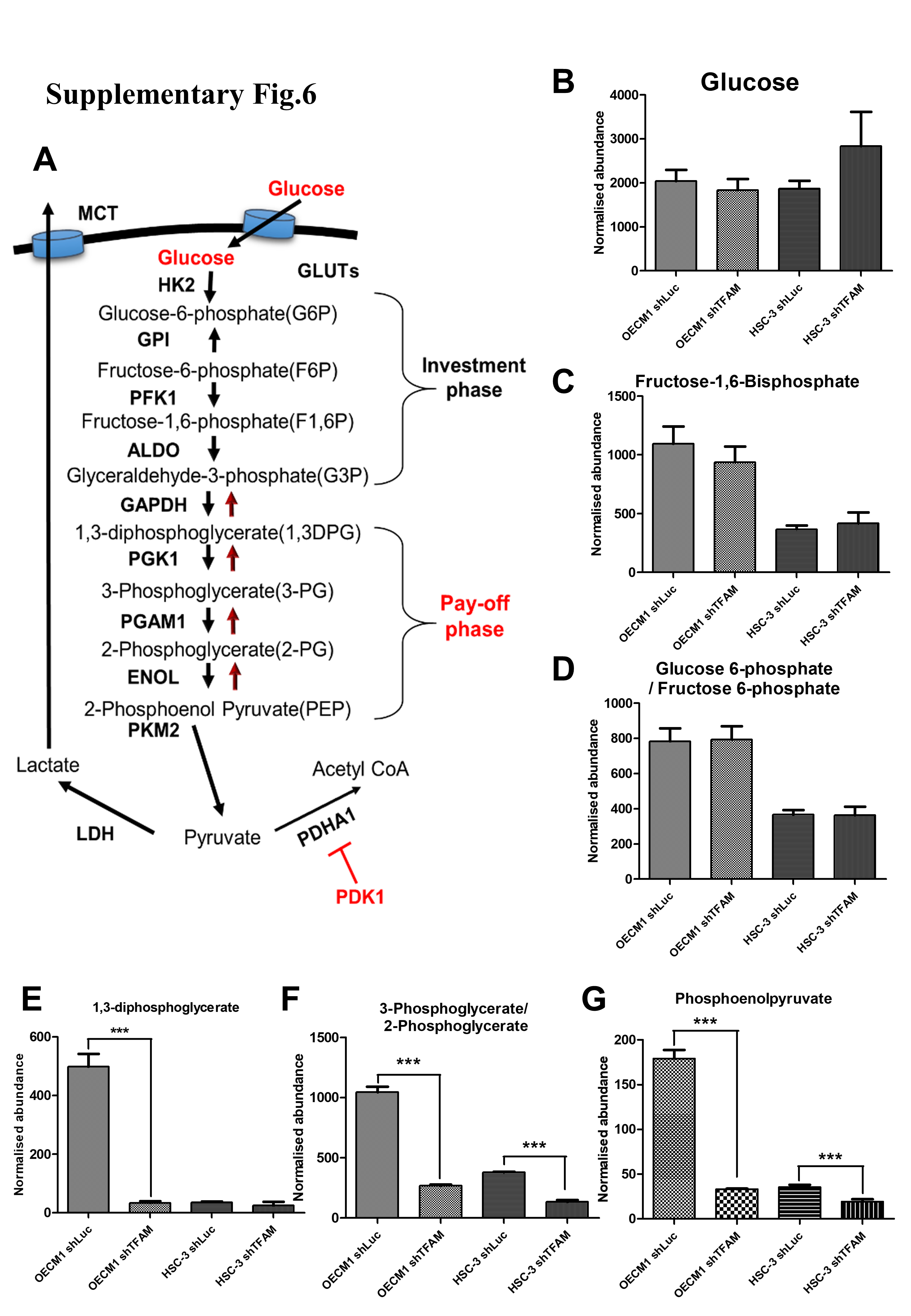

Supplement: Supplementary file 10 — Supplementary Fig.6 [file 41419_2021_4255_MOESM10_ESM.tif]

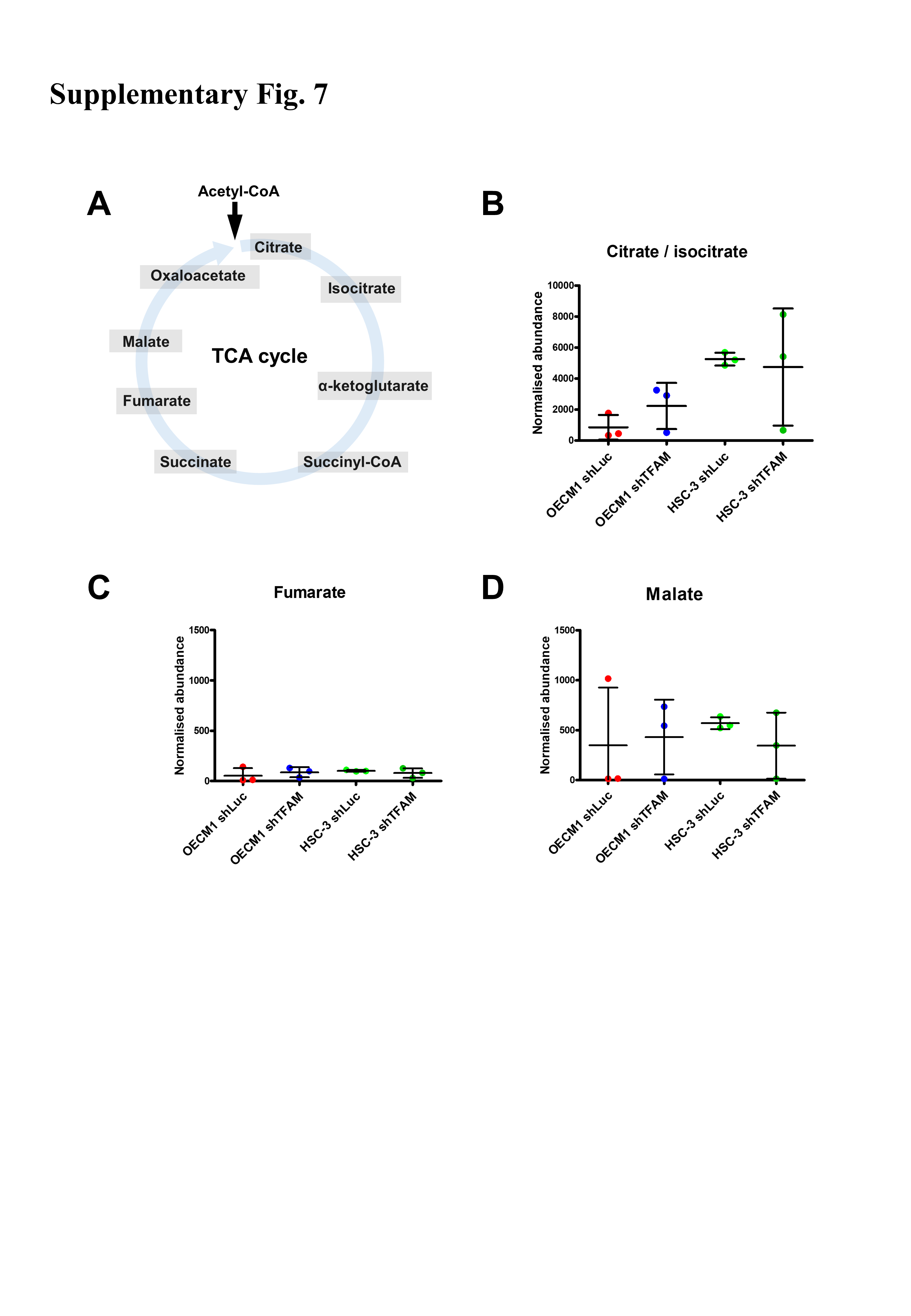

Supplement: Supplementary file 11 — Supplementary Fig.7 [file 41419_2021_4255_MOESM11_ESM.tif]

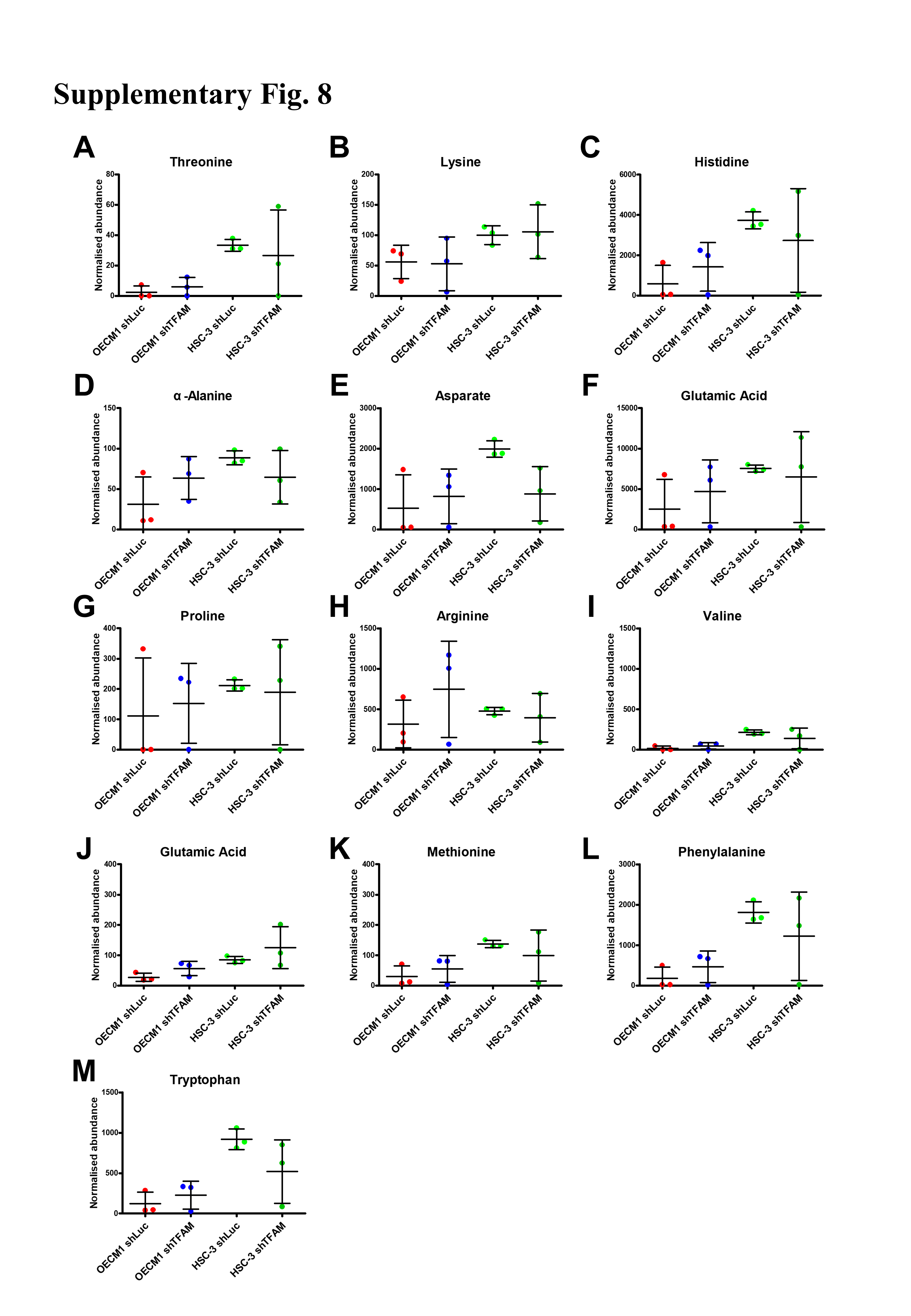

Supplement: Supplementary file 12 — Supplementary Fig.8 [file 41419_2021_4255_MOESM12_ESM.tif]

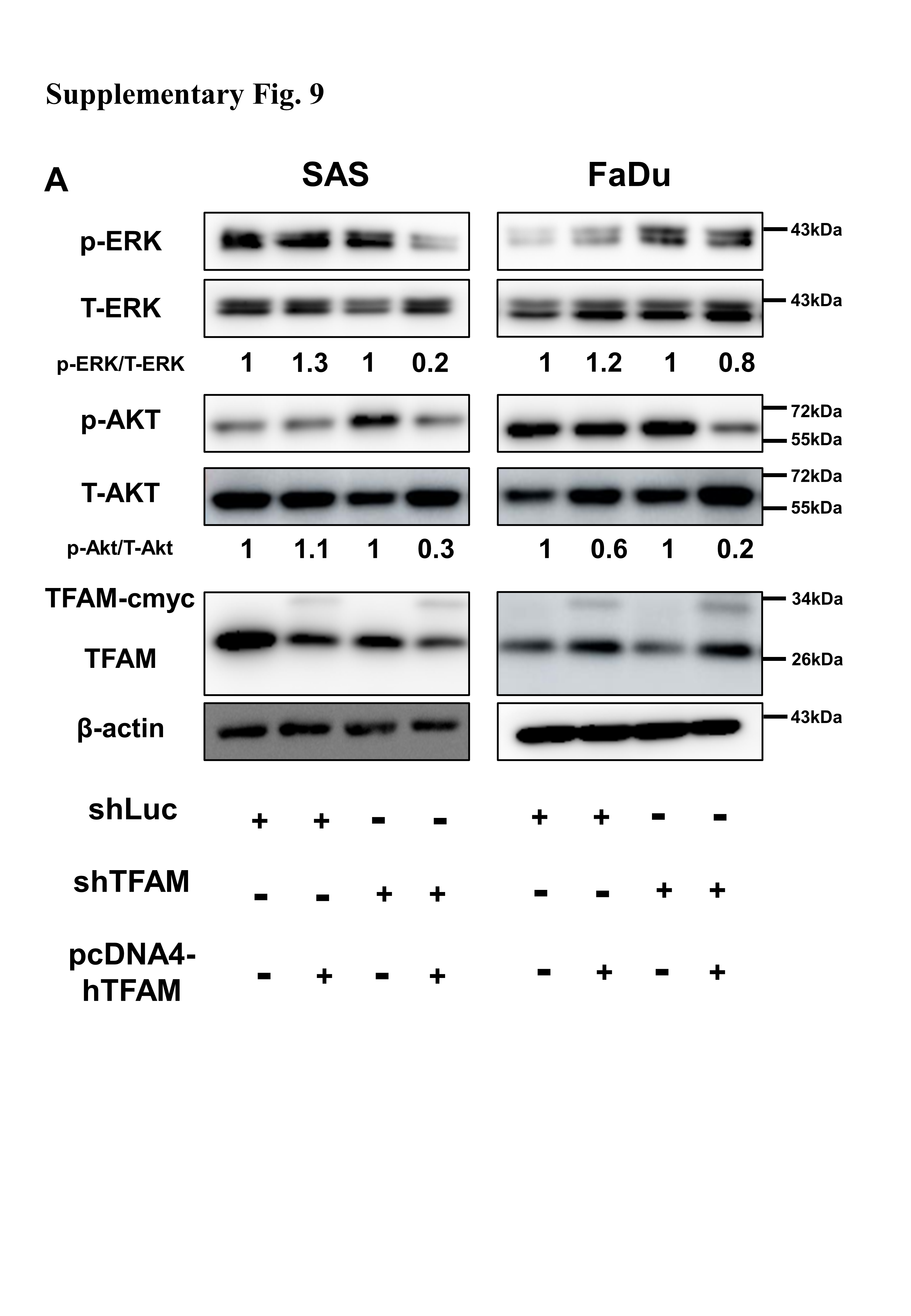

Supplement: Supplementary file 13 — Supplementary Fig.9 [file 41419_2021_4255_MOESM13_ESM.tif]

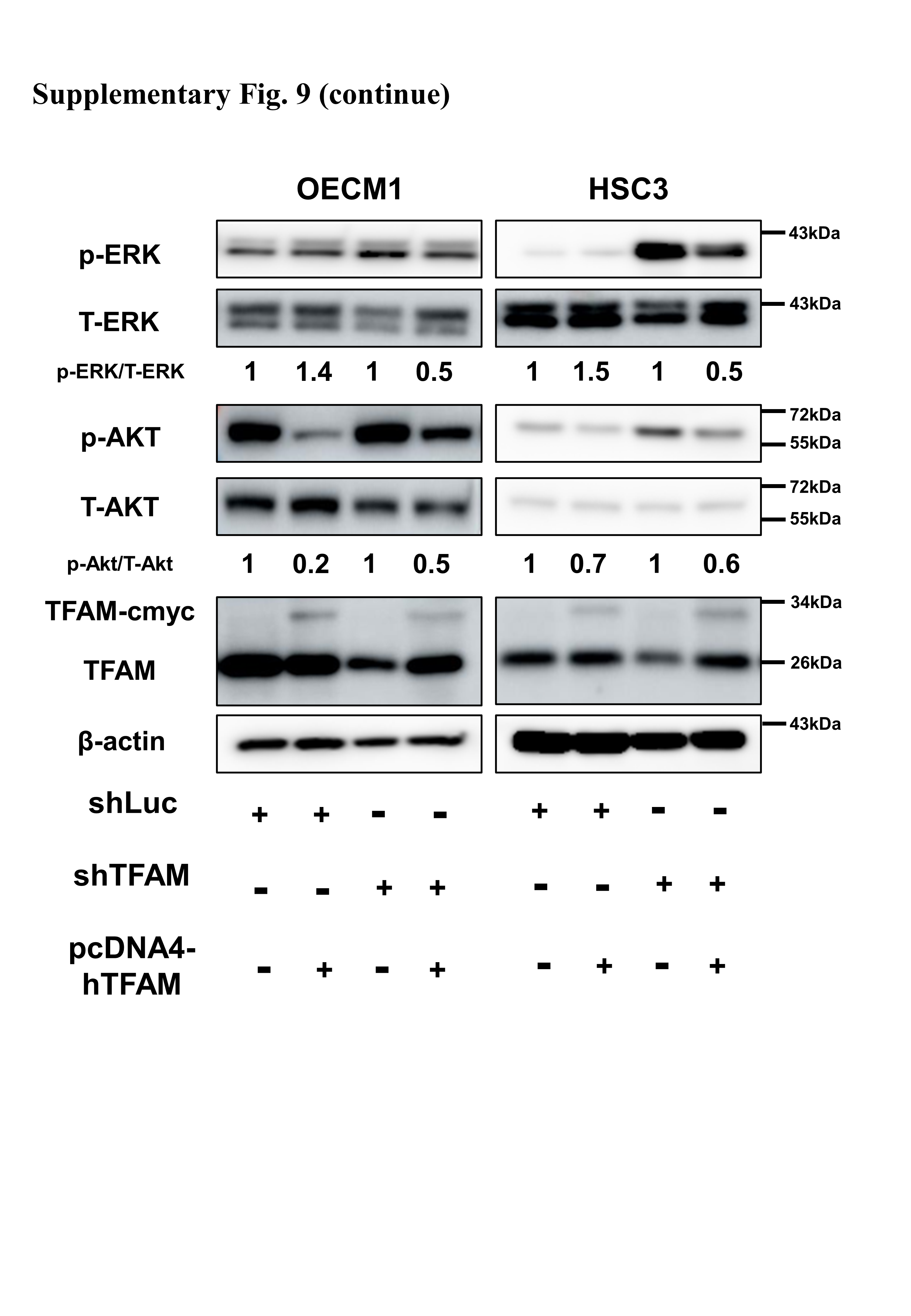

Supplement: Supplementary file 14 — Supplementary Fig.9 (Continue) [file 41419_2021_4255_MOESM14_ESM.tif]

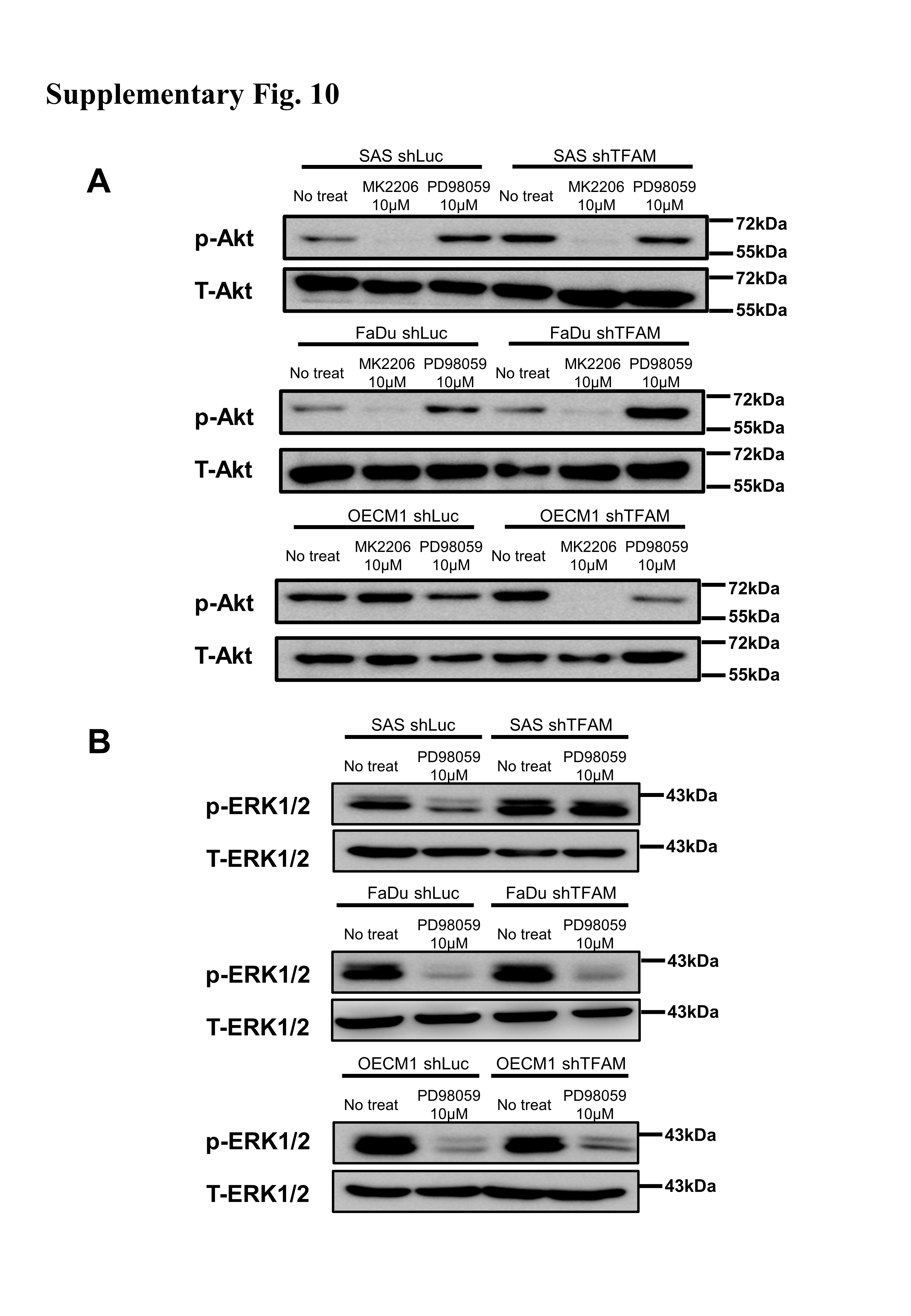

Supplement: Supplementary file 15 — Supplementary Fig.10 [file 41419_2021_4255_MOESM15_ESM.tif]

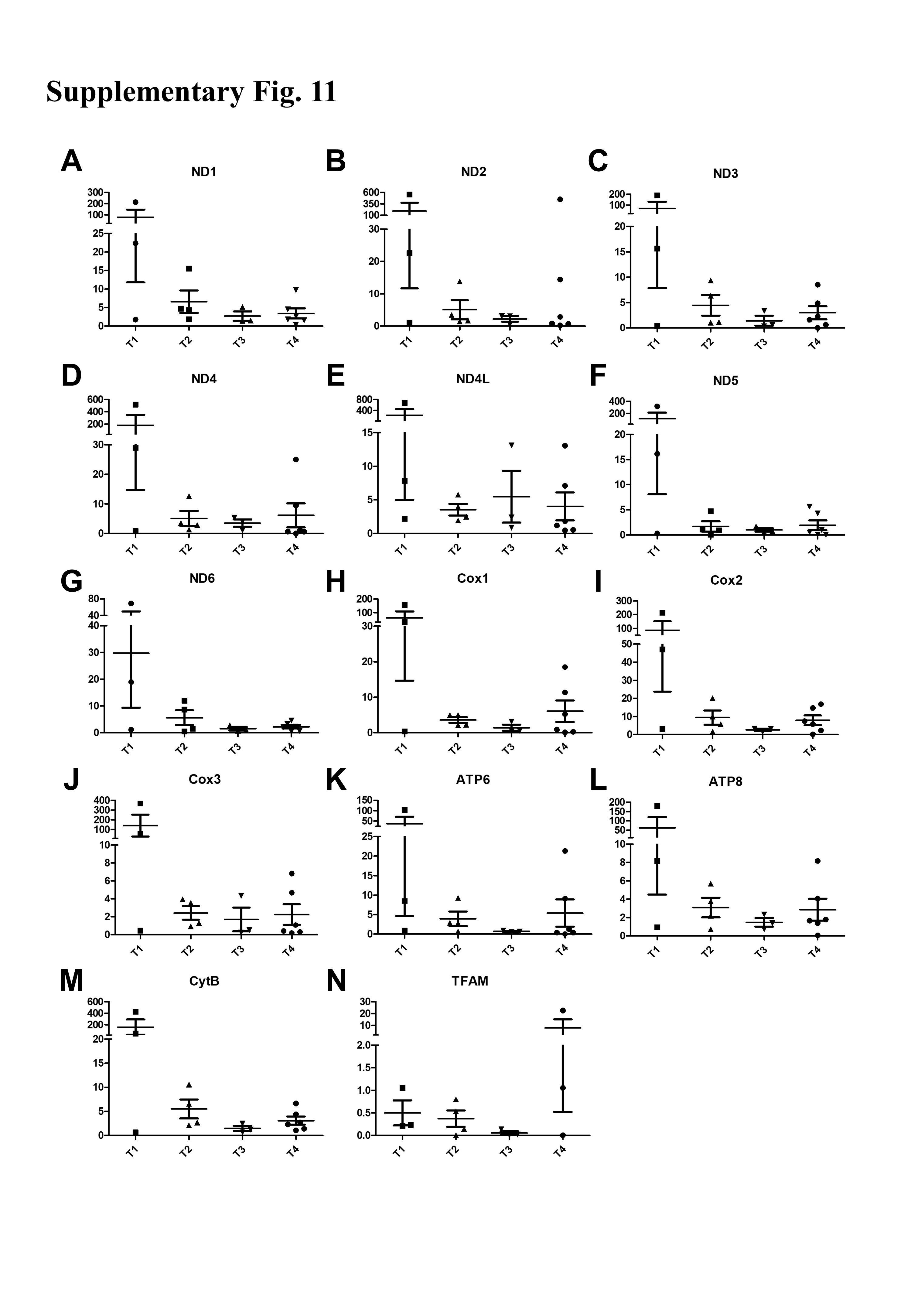

Supplement: Supplementary file 16 — Supplementary Fig.11 [file 41419_2021_4255_MOESM16_ESM.tif]

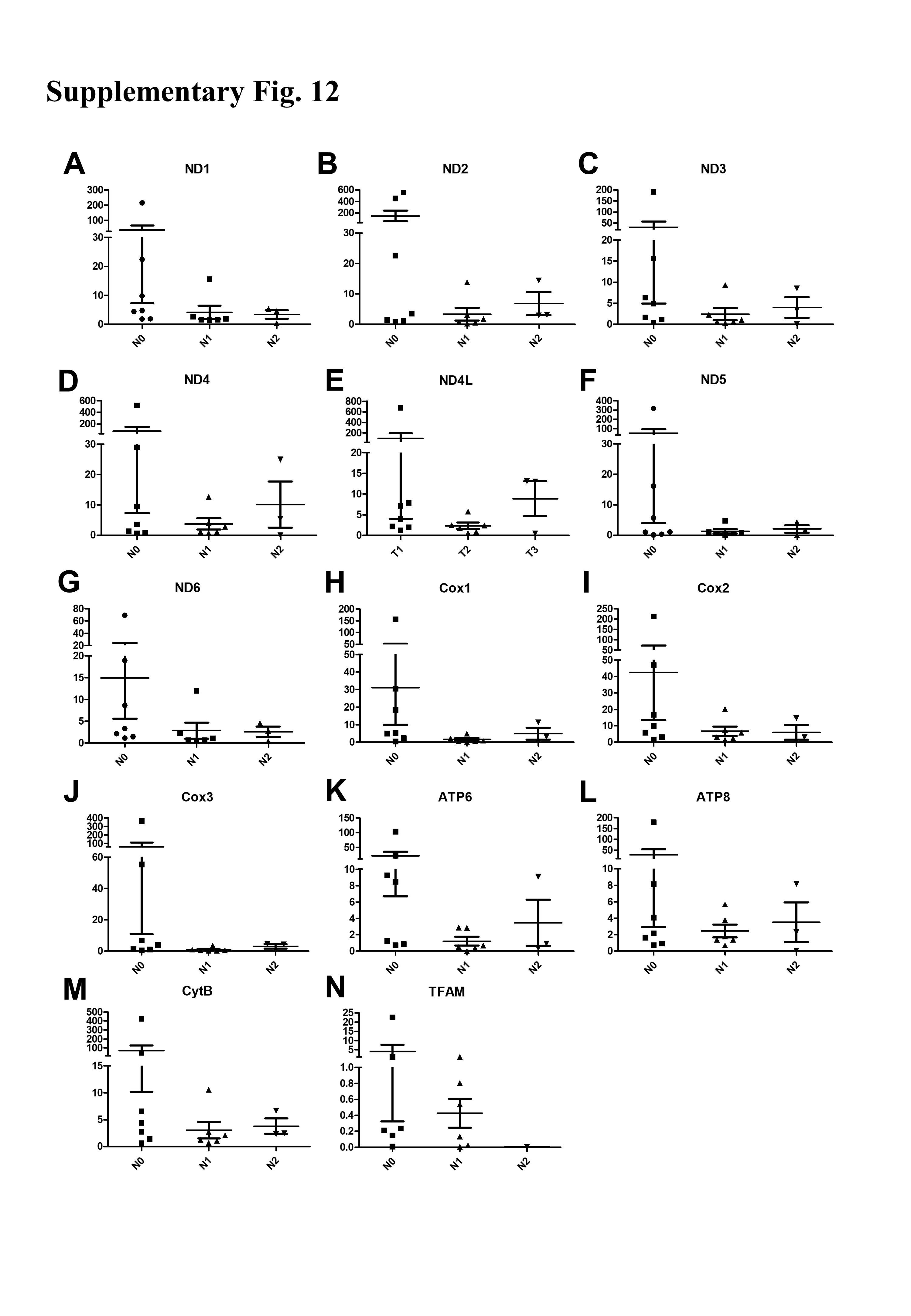

Supplement: Supplementary file 17 — Supplementary Fig.12 [file 41419_2021_4255_MOESM17_ESM.tif]
