# Supplementary material for: Mitochondrial genome and its regulator TFAM modulates head and neck tumourigenesis through intracellular metabolic reprogramming and activation of oncogenic effectors
Source: Cell Death Dis. 2021 Oct 18;12(11):961. doi: 10.1038/s41419-021-04255-w (PMC8523524; doi:10.1038/s41419-021-04255-w)
Supplement: Supplementary file 18 — Supplementary Table 1-6 [file 41419_2021_4255_MOESM18_ESM.pptx]

## Slide 1
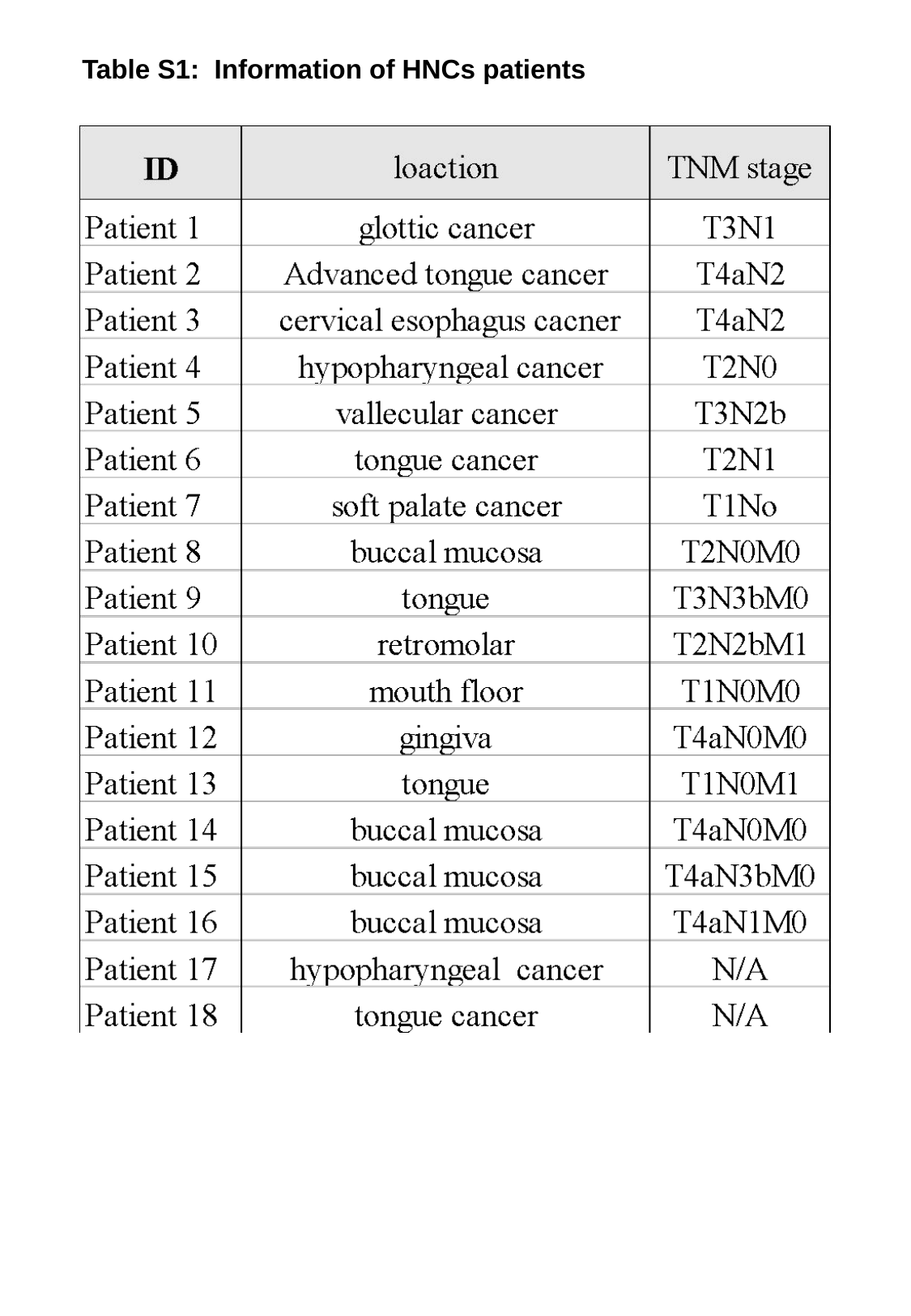

Table S1: Information of HNCs patients

## Slide 2
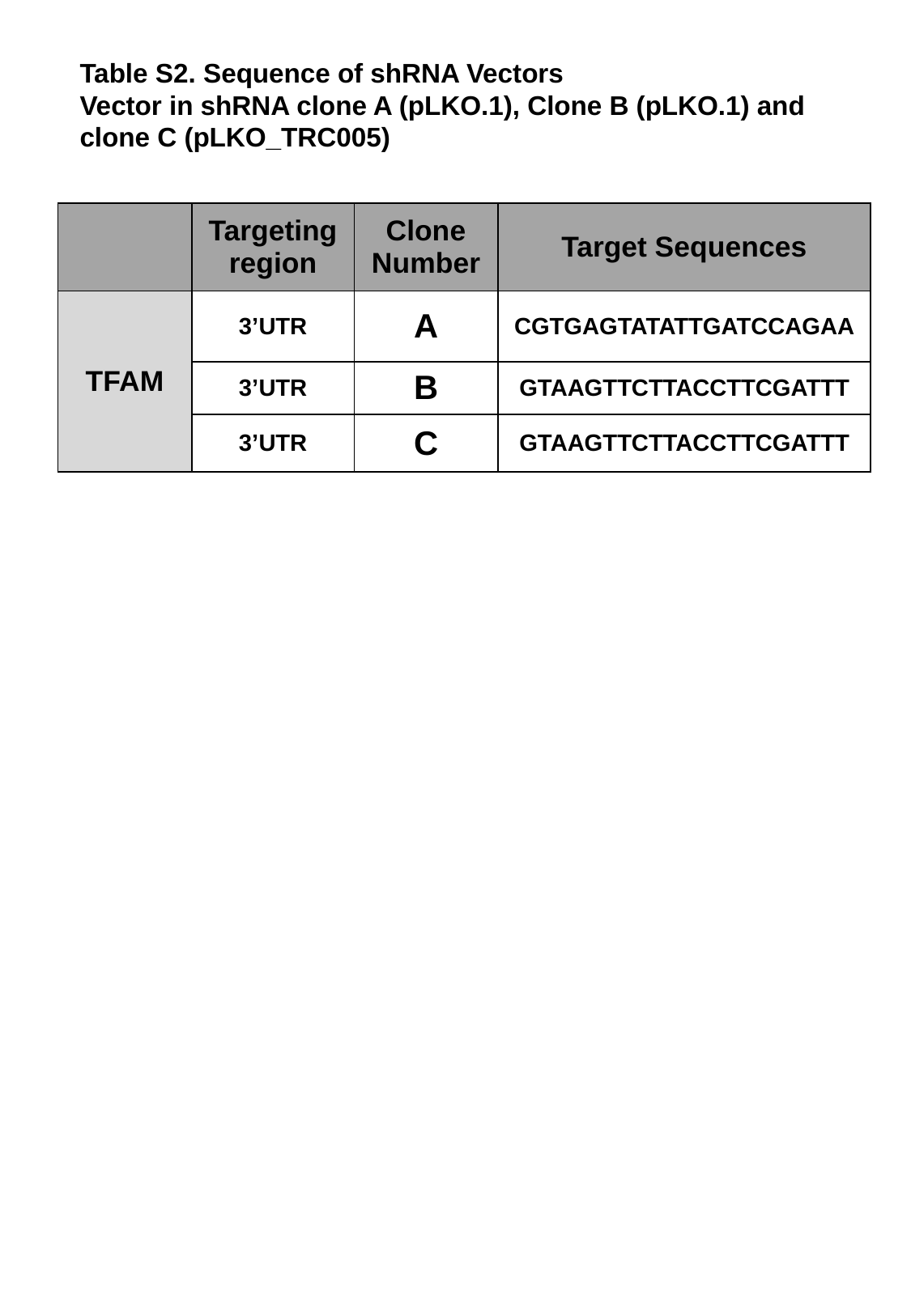

Table S2. Sequence of shRNA Vectors
Vector in shRNA clone A (pLKO.1), Clone B (pLKO.1) and clone C (pLKO_TRC005)
| | Targeting region | Clone Number | Target Sequences |
| --- | --- | --- | --- |
| TFAM | 3’UTR | A | CGTGAGTATATTGATCCAGAA |
| | 3’UTR | B | GTAAGTTCTTACCTTCGATTT |
| | 3’UTR | C | GTAAGTTCTTACCTTCGATTT |

## Slide 3
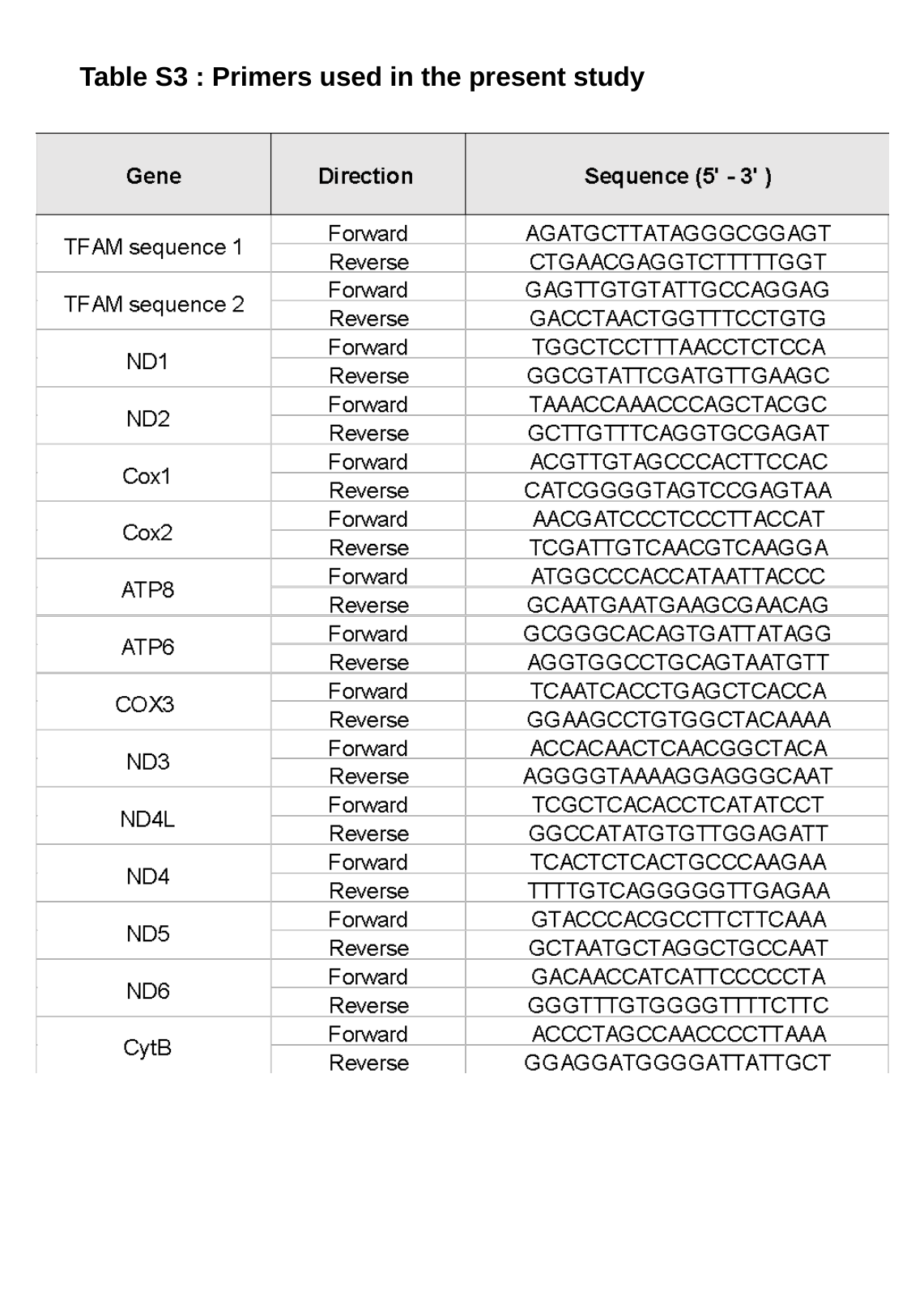

Table S3 : Primers used in the present study

## Slide 4
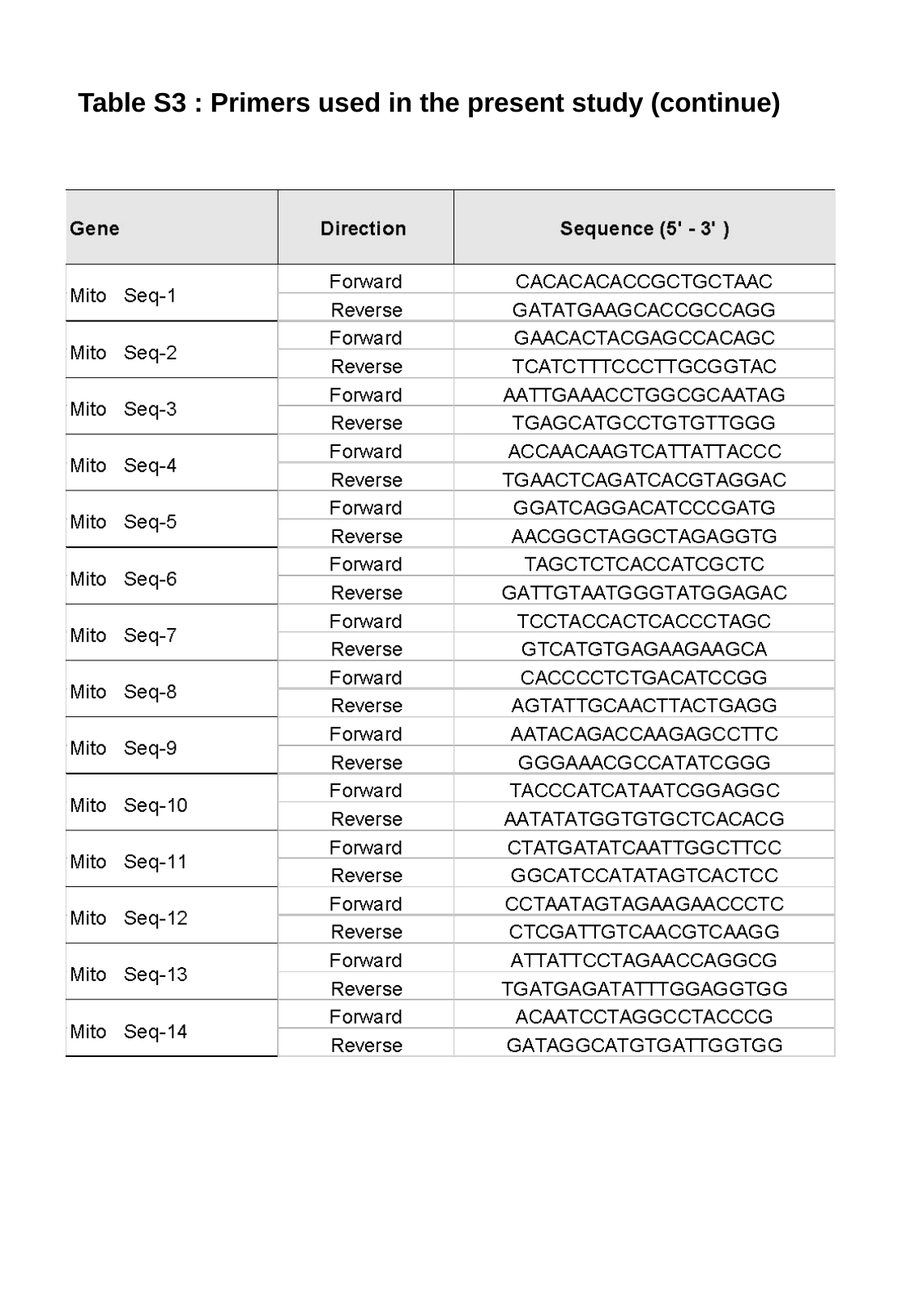

Table S3 : Primers used in the present study (continue)

## Slide 5
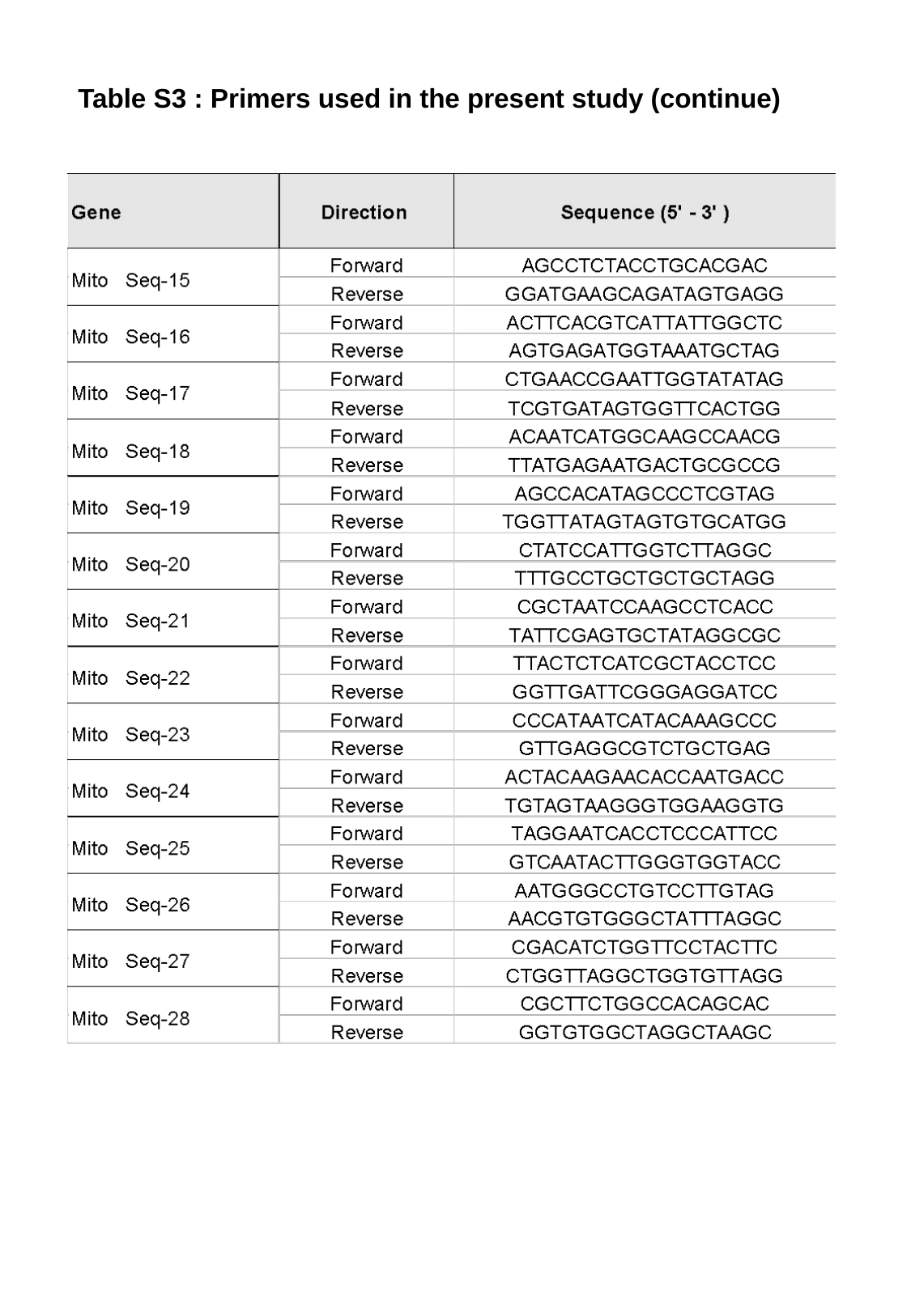

Table S3 : Primers used in the present study (continue)

## Slide 6
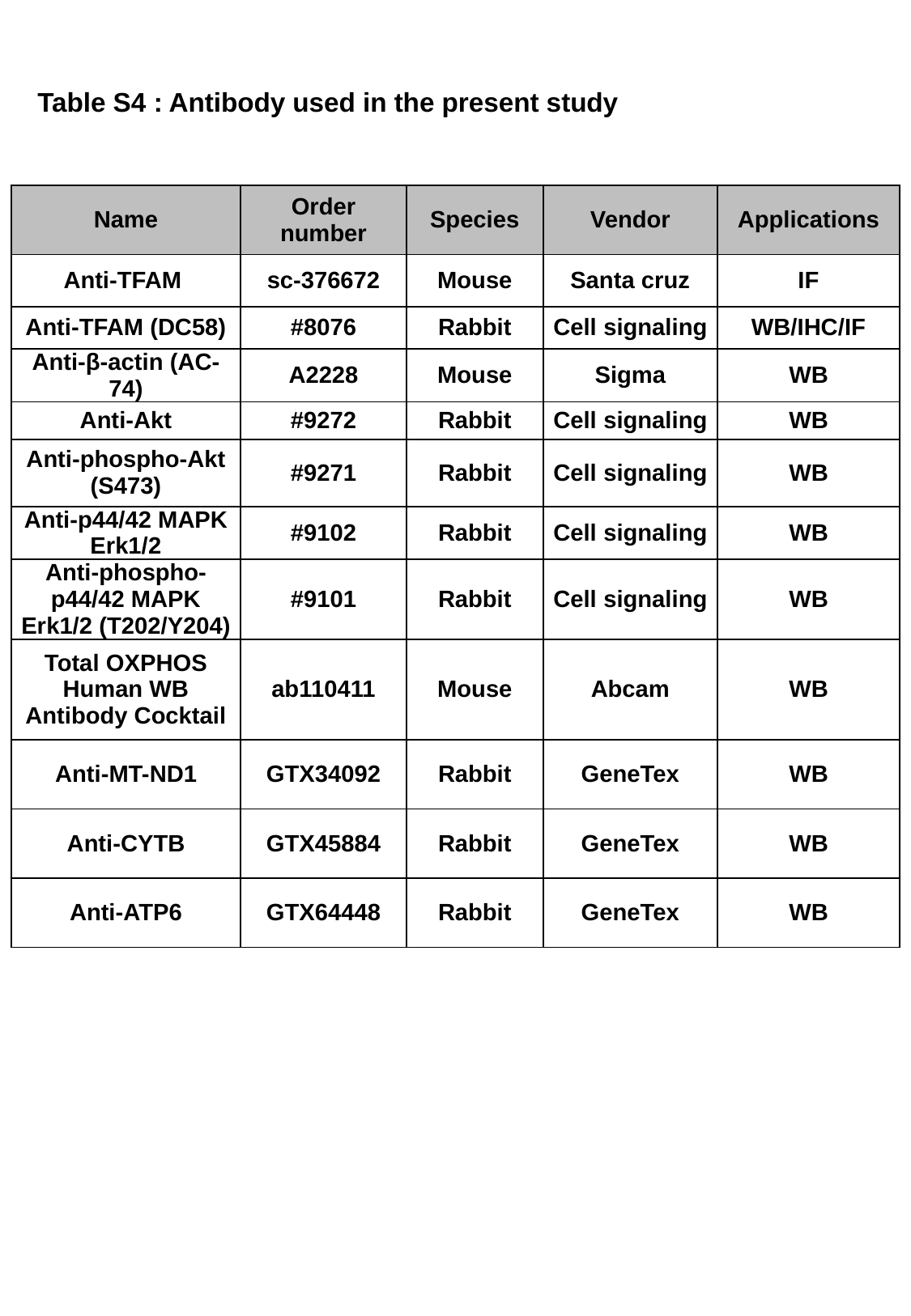

Table S4 : Antibody used in the present study
| Name | Order number | Species | Vendor | Applications |
| --- | --- | --- | --- | --- |
| Anti-TFAM | sc-376672 | Mouse | Santa cruz | IF |
| Anti-TFAM (DC58) | #8076 | Rabbit | Cell signaling | WB/IHC/IF |
| Anti-β-actin (AC-74) | A2228 | Mouse | Sigma | WB |
| Anti-Akt | #9272 | Rabbit | Cell signaling | WB |
| Anti-phospho-Akt (S473) | #9271 | Rabbit | Cell signaling | WB |
| Anti-p44/42 MAPK Erk1/2 | #9102 | Rabbit | Cell signaling | WB |
| Anti-phospho-p44/42 MAPK Erk1/2 (T202/Y204) | #9101 | Rabbit | Cell signaling | WB |
| Total OXPHOS Human WB Antibody Cocktail | ab110411 | Mouse | Abcam | WB |
| Anti-MT-ND1 | GTX34092 | Rabbit | GeneTex | WB |
| Anti-CYTB | GTX45884 | Rabbit | GeneTex | WB |
| Anti-ATP6 | GTX64448 | Rabbit | GeneTex | WB |

## Slide 7
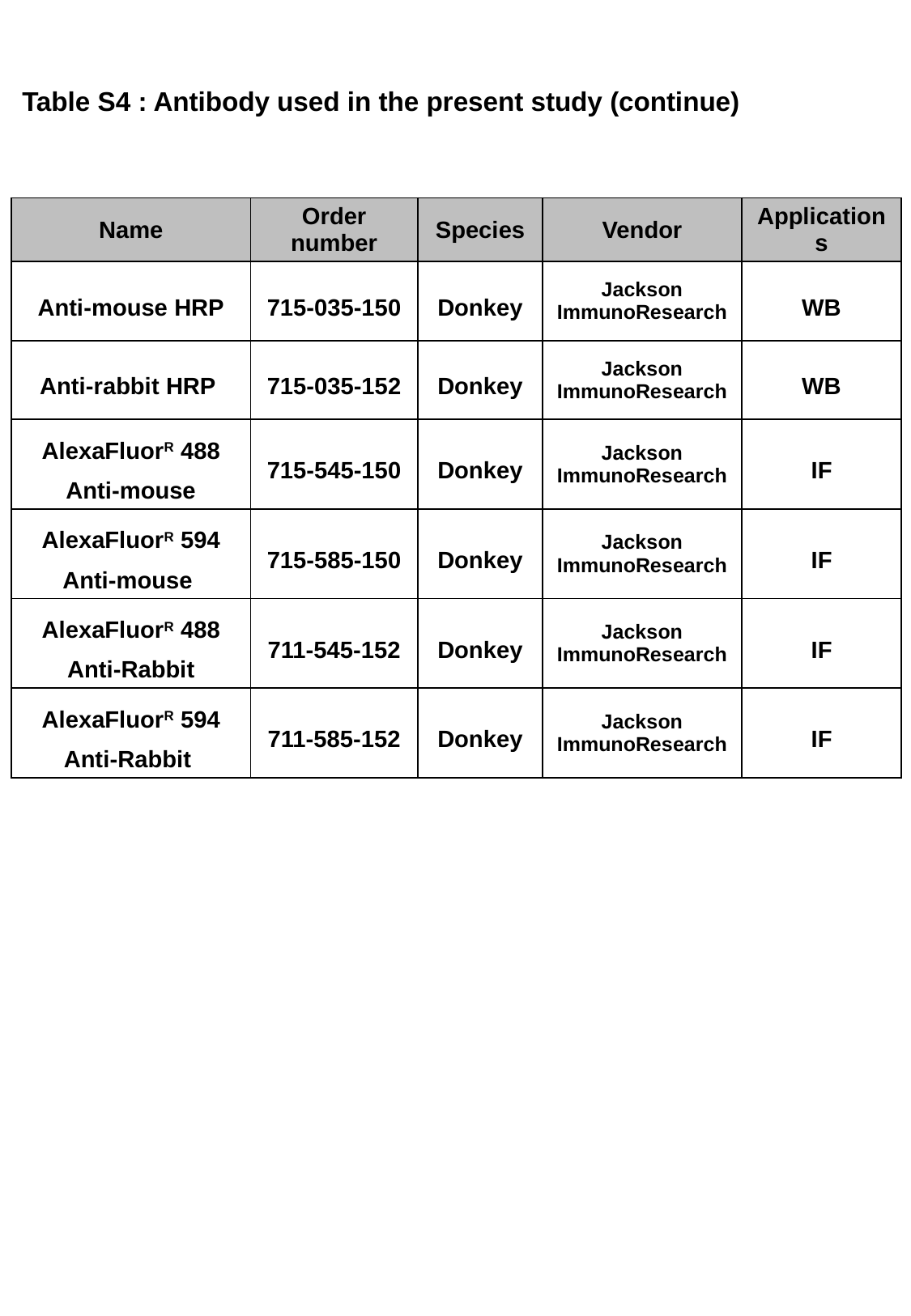

Table S4 : Antibody used in the present study (continue)
| Name | Order number | Species | Vendor | Applications |
| --- | --- | --- | --- | --- |
| Anti-mouse HRP | 715-035-150 | Donkey | Jackson ImmunoResearch | WB |
| Anti-rabbit HRP | 715-035-152 | Donkey | Jackson ImmunoResearch | WB |
| AlexaFluorR 488 Anti-mouse | 715-545-150 | Donkey | Jackson ImmunoResearch | IF |
| AlexaFluorR 594 Anti-mouse | 715-585-150 | Donkey | Jackson ImmunoResearch | IF |
| AlexaFluorR 488 Anti-Rabbit | 711-545-152 | Donkey | Jackson ImmunoResearch | IF |
| AlexaFluorR 594 Anti-Rabbit | 711-585-152 | Donkey | Jackson ImmunoResearch | IF |

## Slide 8
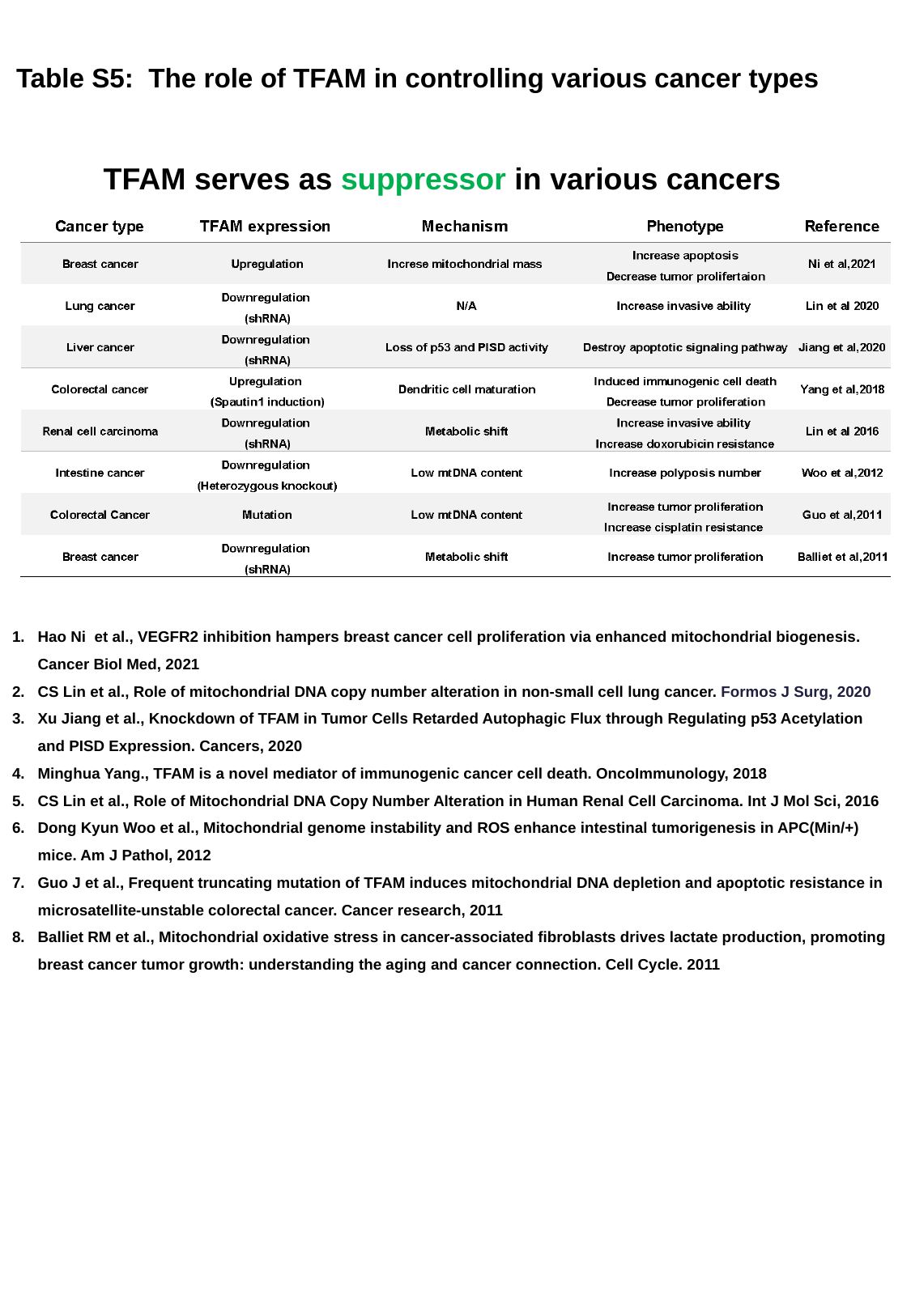

Table S5: The role of TFAM in controlling various cancer types
TFAM serves as suppressor in various cancers
Hao Ni et al., VEGFR2 inhibition hampers breast cancer cell proliferation via enhanced mitochondrial biogenesis. Cancer Biol Med, 2021
CS Lin et al., Role of mitochondrial DNA copy number alteration in non-small cell lung cancer. Formos J Surg, 2020
Xu Jiang et al., Knockdown of TFAM in Tumor Cells Retarded Autophagic Flux through Regulating p53 Acetylation and PISD Expression. Cancers, 2020
Minghua Yang., TFAM is a novel mediator of immunogenic cancer cell death. OncoImmunology, 2018
CS Lin et al., Role of Mitochondrial DNA Copy Number Alteration in Human Renal Cell Carcinoma. Int J Mol Sci, 2016
Dong Kyun Woo et al., Mitochondrial genome instability and ROS enhance intestinal tumorigenesis in APC(Min/+) mice. Am J Pathol, 2012
Guo J et al., Frequent truncating mutation of TFAM induces mitochondrial DNA depletion and apoptotic resistance in microsatellite-unstable colorectal cancer. Cancer research, 2011
Balliet RM et al., Mitochondrial oxidative stress in cancer-associated fibroblasts drives lactate production, promoting breast cancer tumor growth: understanding the aging and cancer connection. Cell Cycle. 2011

## Slide 9
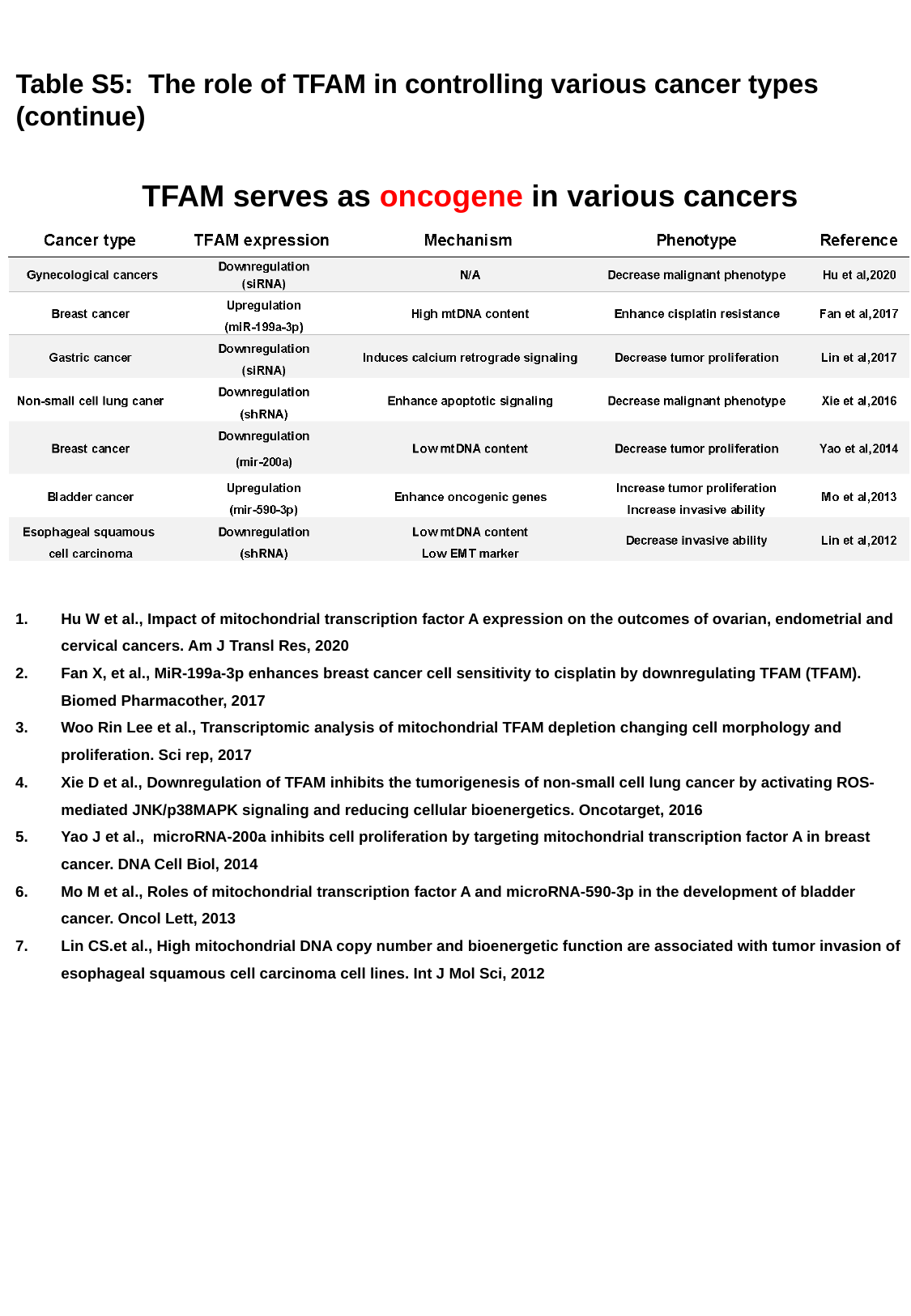

Table S5: The role of TFAM in controlling various cancer types (continue)
TFAM serves as oncogene in various cancers
Hu W et al., Impact of mitochondrial transcription factor A expression on the outcomes of ovarian, endometrial and cervical cancers. Am J Transl Res, 2020
Fan X, et al., MiR-199a-3p enhances breast cancer cell sensitivity to cisplatin by downregulating TFAM (TFAM). Biomed Pharmacother, 2017
Woo Rin Lee et al., Transcriptomic analysis of mitochondrial TFAM depletion changing cell morphology and proliferation. Sci rep, 2017
Xie D et al., Downregulation of TFAM inhibits the tumorigenesis of non-small cell lung cancer by activating ROS-mediated JNK/p38MAPK signaling and reducing cellular bioenergetics. Oncotarget, 2016
Yao J et al., microRNA-200a inhibits cell proliferation by targeting mitochondrial transcription factor A in breast cancer. DNA Cell Biol, 2014
Mo M et al., Roles of mitochondrial transcription factor A and microRNA-590-3p in the development of bladder cancer. Oncol Lett, 2013
Lin CS.et al., High mitochondrial DNA copy number and bioenergetic function are associated with tumor invasion of esophageal squamous cell carcinoma cell lines. Int J Mol Sci, 2012

## Slide 10
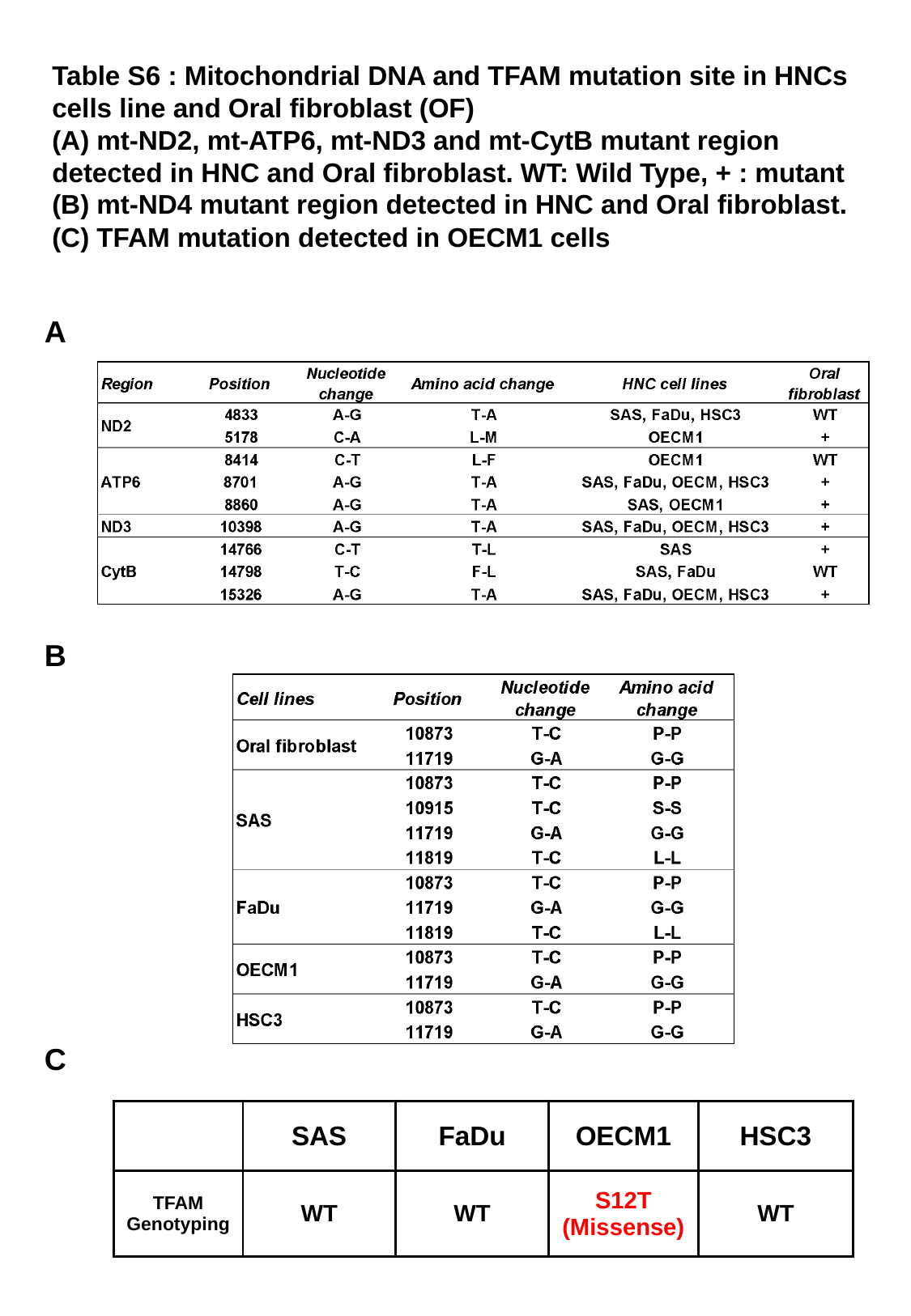

Table S6 : Mitochondrial DNA and TFAM mutation site in HNCs cells line and Oral fibroblast (OF)
(A) mt-ND2, mt-ATP6, mt-ND3 and mt-CytB mutant region detected in HNC and Oral fibroblast. WT: Wild Type, + : mutant
(B) mt-ND4 mutant region detected in HNC and Oral fibroblast.
(C) TFAM mutation detected in OECM1 cells
A
B
C
| | SAS | FaDu | OECM1 | HSC3 |
| --- | --- | --- | --- | --- |
| TFAM Genotyping | WT | WT | S12T (Missense) | WT |
